# Dataset for classifying English words into difficulty levels by undergraduate and postgraduate students

**DOI:** 10.1016/j.dib.2023.109744

**Published:** 2023-10-31

**Authors:** Nisar Ahmad Kangoo, Manmohan Sharma, Apash Roy

**Affiliations:** a School of Computer Science and Engineering, Lovely Professional University, Phagwara Sub-District, Punjab, India; b Department of Computer Science and Engineering, NSHM Knowledge Campus, West Bengal, India

**Keywords:** English language, Difficulty level, Natural language processing, Text classification

## Abstract

The ability of a student to know the meaning of a word depends on the level of education they are at. Since most information is stored in text, students use electronic gadgets to obtain information about their study subjects. This Dataset provides the classification of English words into difficulty levels as viewed by the students at graduation and post-graduation levels. The Dataset in the consideration categorizes the English words into difficulty levels as viewed by undergraduate and postgraduate students in non-native English-speaking countries like India. Some words are neither considered difficult by undergraduate nor postgraduate students. The Dataset can help researchers provide meaning to difficult English words in native languages at runtime (while reading a text document). The Dataset can also help authors write their books and articles for undergraduate and postgraduate levels in different tones, keeping their vocabulary in view.

Specifications Table

**Table:** 

Subject	Computer Science
Specific subject area	English Language, Text Classification, Natural Language processing
Type of data	Text files, CSV Files
How the data were acquired	Data was acquired by the on-spot distribution of questionnaires as hard copy text files and online mode using Google Forms. The questionnaires were prepared from the English textbooks of Indra Gandhi National Open University (IGNOU).
Data format	Raw Analyzed Filtered
Description of data collection	The hard copies of the text files collected have difficult words underlined by students. The data collected provides the number of undergraduate and postgraduate students who marked a word as difficult against each word. The words not marked as difficult by any student have a value of 0. In the case of Google Forms, the students checked the checklist for the difficult words.
Data source location	• Institution: Universities and Colleges • City/Town/Region: Jammu and Kashmir (Union Territory of India). • Country: India • Latitude and Longitude: 32.2778° N, 75.3412° E
Data accessibility	Repository name: Mendeley Data Data identification number: 10.17632/p2wrs7hm4z.4 Direct URL to data: https://data.mendeley.com/datasets/p2wrs7hm4z/4

## 1 Value of the data

• Data can be used to explore the relationship between education level and perception of vocabulary.
• Data will help researchers develop methods for classifying a document into different difficulty levels (text classification purpose).
• The Dataset can help researchers provide meaning to difficult English words in native languages at runtime (while reading a text document).
• The Dataset can also help authors write their books and articles for undergraduate and postgraduate levels in different tones, keeping their vocabulary in view.

## 2 Objective

Language Learning: The Dataset can be used to develop language learning resources or tools that provide exercises or practice materials for learners at different proficiency levels. It can help design vocabulary quizzes, word games, or educational apps focused on building vocabulary skills.

Educational Research: Researchers can analyze the Dataset to gain insights into the linguistic characteristics of words at different difficulty levels. This can be useful for studying language acquisition, cognitive processes involved in word comprehension, or developing linguistic models to understand how difficulty levels impact reading comprehension.

Content Creation: The Dataset can aid in creating educational materials, such as textbooks, graded readers, or online courses, where the difficulty level of the content needs to be carefully calibrated to match the target audience's proficiency level.

Natural Language Processing (NLP): The Dataset can train and evaluate NLP models, such as language models or sentiment analysis algorithms, to handle text with varying difficulty levels. This can help improve language processing tasks, including text classification, sentiment analysis, or machine translation.

Cognitive Science: Researchers studying cognitive processes, memory, or attention can utilize the Dataset to investigate how different difficulty levels impact cognitive load or information processing. It can provide valuable insights into how humans perceive, process, and recall words of varying complexity.

Speech and Language Therapy: The Dataset can assist in developing resources for individuals with speech or language difficulties. Speech therapists can use it to create exercises, practice materials, or speech recognition systems that adapt to different difficulty levels and help individuals improve their language skills.

## 3 Data Description

The basic units of Natural Language Processing (NLP) as mathematical and computational modelling [1] are the words [2]. In NLP, a corpus is a collection of pieces of language text in electronic form selected to represent a language [3]. The language text is chopped into segments known as tokens or words. In English, text can easily be tokenized because there is a blank space between two tokens or words [2]. The Dataset is a CSV format file (dataset_english.csv) where the 2nd column is an English Language word. These words are from the text files or Google form data circulated for data collection. The texts in these files have been taken from Indra Gandhi National Open University (IGNOU) English textbooks for graduate and postgraduate students.

The other columns in this CSV file contain features of these words. The “fre” column tells us how often the word was present in all the text files. Column “len” gives us the length of the word or string in the first column. The “ps” column tells us about the part of the speech feature of the word, whether it is a noun, adjective, verb or adverb. Python code “nltk.pos_tag” populated the ”ps” field with Part of Speech for each word. Columns “difficult_ug” and “difficult_pg” indicate the number of undergraduate and postgraduate students marked a particular word as difficult. Zero (0) in these two columns means the word is not marked as difficult by undergraduate or postgraduate students. This CSV file contains data as below in Table 1.

**Table 1:** The CSV file shows a glimpse of a few rows

Word	fre	len	ps	difficult_ug	difficult_pg
Mire	1	4	(‘mire’, ‘NN’)	-	3
Neck	1	4	(‘neck’, ‘NN’)	-	0
twirling	1	4	(‘twirling’, ‘VBG’)	20	-
food	1	4	(‘none’, ‘NN’)	0	0

Another file in the repository is data_numerical.csv. This file contains all the fields in the above CSV file and numerical values for the “ps” and “word” fields. Since the “ps” and “word” fields are not numerical values, these are converted into numerical form by using the LabelEncoder method in Python. The shape of the file is depicted in Table 2.

**Table 2:** The CSV file shows a few rows with numerical values for POS and word fields.

Word	fre	len	Ps	difficult_ug	difficult_pg	POS_n	words_n
A	784	1	(‘a’, ‘DT’)	0	0	2	2
I	51	1	(‘i’, ‘NN’)	0	0	2311	2315
Am	2	2	(‘am’, ‘VBP’)	0	-	188	188
Go	9	2	(‘go’, ‘VB’)	0	0	2052	2055
Me	12	2	(‘me’, ‘PRP’)	0	0	2928	2928
My	20	2	(‘my’, ‘PRP$’)	0	0	3111	3111
Us	29	2	(‘us’, ‘PRP’)	0	0	5086	5086
If	32	2	(‘if’, ‘IN’)	0	0	2327	2327

The other files uploaded to the repository are:

The English language difficulty level measurement –Questionnaire1.docx
The English language difficulty level measurement –Questionnaire2.docx
The English language difficulty level measurement –Questionnaire3.docx
The English language difficulty level measurement –Questionnaire4.docx
The English language difficulty level measurement –Questionnaire5.docx
The English language difficulty level measurement –Questionnaire6.docx
The English language difficulty level measurement –QuestionnairePG1.docx
The English language difficulty level measurement –QuestionnairePG2.docx
The English language difficulty level measurement –QuestionnairePG3.docx
The English language difficulty level measurement –QuestionnairePG4.docx
and IGNOU English zip file

The contents of these .docx files can be viewed using open-source tools such as LibreOffice.

The English language difficulty level measurement -Questionnaire (1-6) & PG1, PG2, PG3, PG4 .docx (10 files) files contains the questionnaire supplied to students of College and University to underline difficult words in the English text. The paragraphs were chosen so that 1^st^-semester graduation students were asked to mark difficult words from the 2^nd^-semester English book. Likewise, every student was asked to mark difficult words from the text selected from the next higher semester. The total number of participants was 960; 193 were pursuing post-graduation, and 767 were pursuing under-graduation.

IGNOU English.zip file contains the Indra Gandhi National Open University (IGNOU) English textbooks for graduation and post-graduation students. The text for the above questionnaires was taken from these IGNOU English textbooks.

## 4 Experimental Design, Materials and Methods

### 4.1 Participants

The data has been collected from university and college students of Jammu and Kashmir (Union Territory) in India. The union territory has nine universities and about 142 degree colleges. The number of participating students was 960; 193 were pursuing post-graduation, and 767 were pursuing under-graduation from universities and colleges, respectively. The participants were 18 to 23 years [4]. The semester-wise details of the participants are given in Table 3

**Table 3:** The semester-wise details of the participants.

Semester	No. of participants
Undergraduate 1^st^ semester	72
Undergraduate 2^nd^ semester	304
Undergraduate 3^rd^ semester	60
Undergraduate 4^th^ semester	103
Undergraduate 5^th^ semester	122
Undergraduate 6^th^ semester	106
Postgraduate 1^st^ semester	52
Postgraduate 2^nd^ semester	54
Postgraduate 3^rd^ semester	37
Postgraduate 4^th^ semester	50
Total	960

### 4.2 Material

**Fig. 1:**
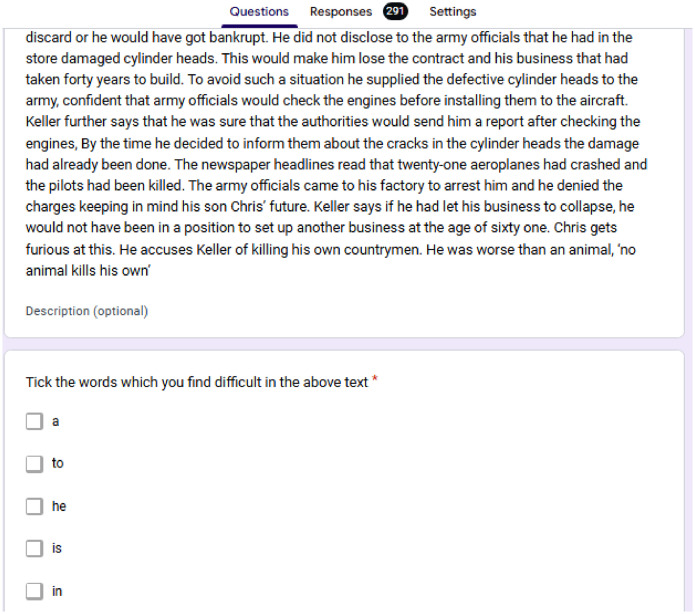
Snapshot of a questionnaire for data collection.

The students were provided hard copies of questionnaires for on-spot labelling of difficult words. The Google forms containing text and all words in the text as checkbox lists were also circulated among students. There were 10 Google forms, with each form having ten text paragraphs. Each paragraph was followed by its words as checkboxes for participants to select the difficult words, as shown in Fig. 1. The Google forms (Questionnaires) were made such that the student of 1^st^ semester was asked to choose difficult words from text provided by the 2^nd^-semester English book. Likewise, every student was asked to select difficult words from the text of the next or higher semester. The texts in these questionnaires were taken from the Indra Gandhi National Open University (IGNOU) undergraduate and postgraduate English Books. The hyperlink for one of the Google forms (Questionnaires) is https://forms.gle/TX5nJptqymuseMop9. The IGNOU is the leading public central university in India, which has study centres across the globe. The Indira Gandhi National Open University (IGNOU) was established by an Act of the Indian Parliament in 1985 [5].

### 4.3 Procedure

According to Webster's Dictionary, a native speaker is a person who learned to speak the language of the place where he or she was born rather than learning it as a foreign language [6]. English is not the 1^st^ language of 96.71% of Indians as per the census 2011 [7]. India has 22 scheduled languages viz. (1) Assamese, (2) Bengali, (3) Gujarati, (4) Hindi, (5) Kannada, (6) Kashmiri, (7) Konkani, (8) Malayalam, (9) Manipuri, (10) Marathi, (11) Nepali, (12) Oriya, (13) Punjabi, (14) Sanskrit, (15) Sindhi, (16) Tamil, (17) Telugu, (18) Urdu (19) Bodo, (20) Santhali, (21) Maithili and (22) Dogri. These scheduled languages do not include the English language. As per the Indian 2011 census, 96.71% of the Indian population speaks these scheduled languages. In the region (Jammu & Kashmir UT) from where the Dataset was collected, 97.27% speak the scheduled Indian language. As per the 2011 census, there are 259678 English speakers, which covers 0.02 percent of India's population [8]. The percentage of English-speaking people in the region where the data was collected is just 0.37%. Thus, we can say the speakers of the area where the data was collected are non-native English speakers. The medium of education in Jammu & Kashmir (UT), as per New Education Policy 2020, should be a mother or home language up to 5^th^ standard and may even be extended to 8^th^ standard [9]. Higher education institutions must also use the mother tongue as a medium of education in addition to English [10].

The data collected via (hard copies or Google forms) was processed. The whole text from all 100 text files (paragraphs from IGNOU English books) was combined and made into a single file. This file was preprocessed by removing punctuation marks and special characters [11]. The remaining words in the text file were converted into the table where a separate row represents each word. This table was copied in MS Excel, where word frequency was calculated using Count Formula. The duplicate words were then removed using the data remove function from the data validation tab in MS Excel. Each word's length was calculated using the length formula in MS Excel. The ability of students to recognize words differs considerably at different levels of education, as do the underlined words or selected checkboxes from collected data [12]. Collected data from hardcopies and Google form responses were looked for underlined/selected words by undergraduate and postgraduate students. The number of undergraduate students who marked a word as difficult is mentioned in the “difficult_ug” column, and the number of postgraduate students who marked a word as difficult is mentioned in the “difficult_pg” column. If these two columns have zero value for a particular word, no undergraduate or postgraduate student has marked that word as difficult. The hyphen (-) against a word in column “difficult_ug” or “difficult_pg” means that the word is not present in the text circulated for undergraduate students or postgraduate students, respectively.

This file is saved in CSV format. Python code “nltk.pos_tag” populated the PS field with Part of Speech for each word. Since the “*ps*” and “*word*” fields are not numerical values, these are converted into numerical form using the LabelEncoder method. The other features that can be added to the Dataset are Word entropy (the degree of uncertainty or randomness in its distribution across a dataset), Word origin(it's linguistic roots and historical usage), Word pronunciation(stress patterns and intonation), Word polarity (positive or negative sentiment associated with it), Word sense (different meanings of a word), Word morphology(the internal structure of a word, such as its root, stem, prefix, and suffix), Word complexity(how complex a word is, which can be based on factors such as word length, syllable count, and the number of morphemes), and Word source(source of a word, such as whether it is a loanword, a technical term, or a colloquialism).

**Fig. 2:**
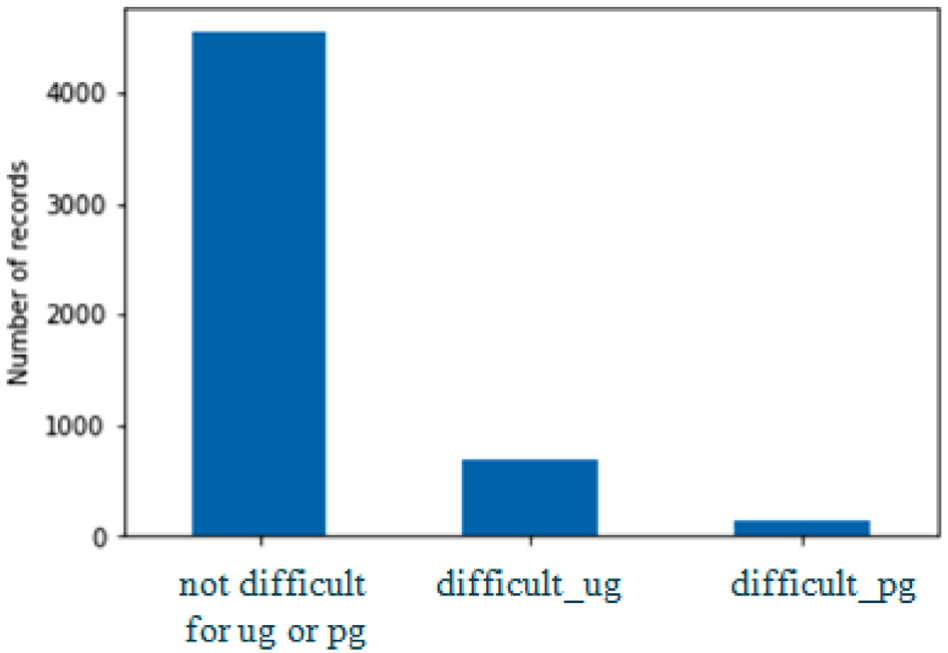
Figure showing a highly imbalanced dataset.

The Dataset has a total of 5368 unique words. The words marked as difficult by undergraduate students are 680, those marked as difficult by postgraduate students are 151, and all remaining words viz 4537 are easy, hence neither marked as difficult by undergraduate students nor by postgraduate students. This Dataset is highly imbalanced because the text under consideration will always have fewer difficult words than easy words. The graph in Fig. 2 shows how the imbalanced data looks.

**Fig. 3:**
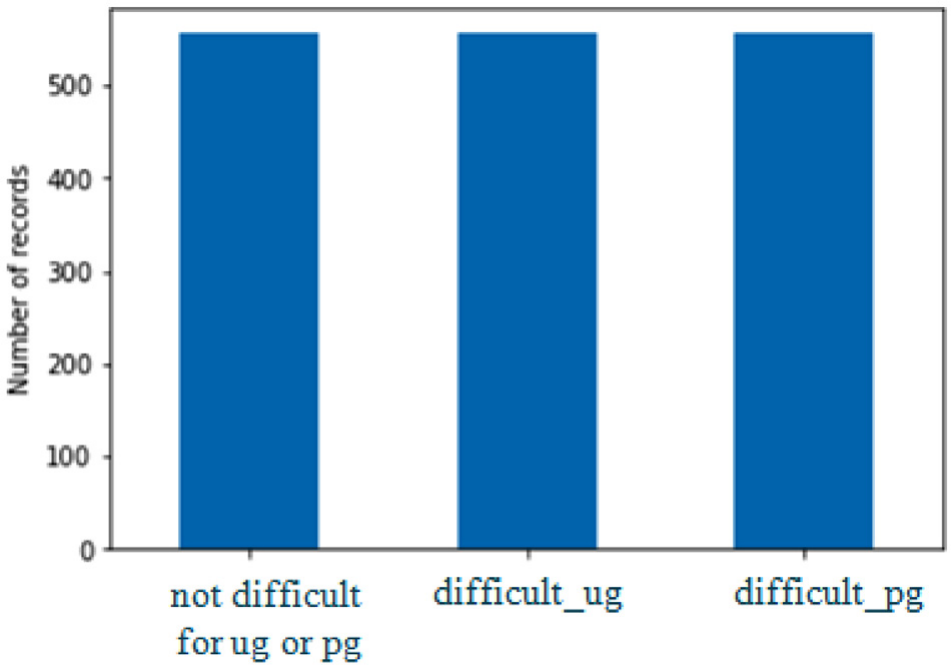
Dataset after using oversampling SMOTE.

The Dataset can be balanced using several balancing techniques, viz under sampling and oversampling, after applying Synthetic Minority Oversampling Technique (SMOTE) from imblearn.over_sampling class in Python, the balanced data looks as shown in Fig. 3.

Although there are several techniques for balancing datasets, we have provided this article with the original Dataset and left using balancing techniques for the users of the Dataset so that they can implement it as per their requirements.

## Ethics Statements

The participants in the survey have voluntarily participated in it. The information collected from the participants has been kept confidential and used only for research proposals. No personal information has been gathered, and the privacy rights of participants have been observed.

## CRediT authorship contribution statement

**Nisar Ahmad Kangoo:** Conceptualization, Methodology, Visualization, Investigation. **Manmohan Sharma:** Data curation, Writing – original draft, Validation, Writing – review & editing. **Apash Roy:** Supervision.

## Declaration of Competing Interest

The authors declare that they have no known competing financial interests or personal relationships which have, or could be perceived to have, influenced the work reported in this article.

## Data Availability

Dataset for classifying English words into difficulty levels by undergraduate and postgraduate students (Original data) (Mendeley Data).

## Appendix Supplementary materials

Image, application 1

Image, application 2

Image, application 3

Image, application 4

Image, application 5

Image, application 6

Image, application 7

Image, application 8

Image, application 9

Image, application 10
