# Supplementary material for: Dataset for classifying English words into difficulty levels by undergraduate and postgraduate students
Source: Data Brief. 2023 Oct 31;51:109744. doi: 10.1016/j.dib.2023.109744 (PMC10661753; doi:10.1016/j.dib.2023.109744)
Supplement: Supplementary file 1 [file mmc1.docx]

The question of language is seen differently by the new writers. As far as they are concerned, the language they write in is theirs. English is their first language. But this is English that has undergone many changes. But even this English cannot represent the complete daily life of any individual in India, where even the diehard

English speaker will have to use some other Indian language(s) in various social situations. If this is not taken into account, many nuances which can be exploited by the writer can be lost to her/him. Also, many Indians do not use English at all in their daily lives. How does the Indian English writer represent them? So even if English is the first language of the new writers, they should be aware of the challenges that have always been there for Indian English writers. The Indian English novel has gone transnational with many writers living in the west and writing from a perspective beyond nationality. On the other hand, though always with the chance of international exposure, there are many novelists who live and publish in India, who still work on questions of identity in this multicultural multilingual nation. This may be the era of the postmodern novel but Indian English novels still do not forsake India.

One day, while at the Collector’s office, Captain hears some villagers complaining to the clerk about the delay in catching the tiger which was fast devouring their cattle. The clerk loses his temper and threatens to call in the police. The villagers walk out of the office followed by Captain who recalls Dadhaji’s injunction that one must ferret out all information if one hears a tiger being mentioned.

Perspective was not the strong point of metaphysical writing. The overall attitude of acceptance or rejection that sifted grain from chaff and took a position was entirely missing from the writings of John Donne, Herbert and many others of the group. In them, there was not much to convey. It might indeed be asked whether poems of these writers led major critics to take note of them. Yet, there was something in the metaphorical verse that drew attention. This may be termed the sense to engage with current thought, whether linked with ideology, religion or popular themes of love and rational appreciation. All these were there in the air. Certain it was that Metaphysical poets did their job of poetically expressing their intent. They had no simple answers to provide to the current issues. What they achieved was in the form of formulating an honest opinion on the questions of the day. For instance, equanimity and balance were missing from the socio-ideological scene at the time. Admitting it as such was an act of courage, and pursuing it intellectually was a challenge worth taking up. What may have suited the environment particularly was a rational appraisal. Thus, intellect was employed to experiment, stretch, dare to seriously unsettle existing norms and show to the reader that all was not well with the period. Since the tangible relations and affinities did not hold much hope, paths untrodden were explored. That they were called not materialist and deeply secular in their involvement might make sense. Yet, the word that captured the essence of this writing was “metaphysical.” This meant beyond the sensible, normal and sane.

As the days pass, the tiger begins to understand what Captain wants of him. His continuous lashings teach him to run round and round, without there being a reason for running, after which he is put back in the cage and given pieces of meat and a trough of water. Thereafter, he is made to learn all sorts of new tricks— he is made to jump over obstacles of all kinds put in his way; he is forced to jump through a ring of fire although he dreads the fire after his past experience when he was nearly roasted by the flaming torches of the villagers; he is made to sit on a stool and sip milk from a saucer placed on a table before him, although the taste of milk nauseates him as he is basically a meat-eater and not a milk-drinker and would gladly have devoured the cow than drink its milk, as he confides to the chimp. Every time the tiger fails to perform a new trick, he is mercilessly lashed with the whip by Captain. As a punishment, he is isolated from other animals and starved for days together till he grows weak and is ready to obey his ring-master. Sometimes he thinks of attacking Captain but the Chair always shields the man. At that time the chair appears like a powerful engine of destruction to the tiger but later when he sees chairs lying still in the schoolroom at Malgudi he realizes that a chair is nothing but a harmless piece of furniture that he could smash in a second, with his paw.

One day, the tiger saw his mate go down the river bank and climb up the other. The cubs somehow escaped his watchful eyes and followed their mother. He realized too late what had happened and followed them. When he heard strange noises around him, he roared to call back his family but in vain. Still following his family, he soon reached human habitation and, to his utter horror, saw his mate and cubs lying dead in a cart being pulled by a line of men singing and dancing around it. The tiger was shattered to the core at the loss of his dear ones. His belief that the jungle was impregnable to human beings was shattered. Anyhow, hiding behind a rock, he saw a set of men arrive in a jeep to take away his dead family. Those days he was untamed and wanted to tear and kill every man present there but somehow held back. The death of his family taught him to take revenge from the village folk by stalking and poaching their cattle for food. Every two days, he took away a sheep from the flock. The villagers did not suspect him for their loss and advanced ignorant theories regarding a tiger’s hunting habits, which amused him a good deal. He now discovered that hunting in the village was far more easy and untiring than the jungle-hunting where the game was more alert and elusive. The villagers, too, discovered they were losing their cattle more regularly and set up an enclosure, with a door, in the centre of the village to trap the culprit. The tiger one day entered the enclosure but as soon as he nabbed a lamb, it’s bleating woke up the villagers who ran out with flaming torches, hatchets and crowbars. In his haste to escape, the tiger lost sight of the door and ran in circles inside the stockade, confused and blinded with the fire from the torches and the frenzied shouting of the villagers.

A certain man in Malgudi, called Captain, had once bought a yellow monkey and a parrot (that could pick up numbers and alphabets from a pack of cards) from an Irishman who earned his living by displaying them in public. Being more ambitious, Captain thought about joining a circus and approached an old man, Dadhaji, who owned “Dadhaji’s Grand Circus” in Poona. Dadhaji wanted to know how much knowledge he had about animals. Captain told him that he had not encountered many animals in his life except the alley cats and mongrels in his street in Malgudi. He disclosed that he had come to Dadhaji to learn about animals and how they were trained. Dadhaji took him in but told him to get rid of the monkey and the parrot that were fit only for street corner shows and not meant for his circus which had around 150 large animals. He told Captain to take charge of cleaning the stables and attending to the horses in return for food, shelter and pocket money. Captain readily accepted the offer and started his circus career under the tutelage of Dadhaji who taught him all about training animals and the business of running a circus, often telling him that there was no wild animal that could not be tamed or trained. When the old man died, Captain inherited the circus- with its property, assets and animals. He soon shifted to his native Malgudi where he set up the “Grand Malgudi Circus” by bribing the authorities in order to overcome their objections to his venture. Malgudi soon became famous for its circus—with its animals, scores of acrobats and performers of all kinds—due to the hard work put in by Captain. Getting up daily at five in the morning he took rounds of the camps to know the welfare of his animals, often making fun of his wife by taunting “….they are tended better than your family.”

The story begins with the aged Raja (The tiger) —who is lying in his cage in a zoo—narrating his life-story. The following words of the narrator, almost at the beginning of the story amply demonstrate this quality:

“You are likely to understand that I am different from the tiger next door and I possess a soul within the forbidding exterior. I can think, analyse, judge, remember, and do everything you do, perhaps with greater subtlety and sense. I lack only the facility of speech. But if you could read my thoughts, you would be welcome to come in and listen to the story of my life.” After persuading the reader in the above manner, the narrator switches back to the past. Raja (The tiger) talks about his days in the Mempi forest where he is the acknowledged master and king of animals. Raja cohabits with a tigress and begets a litter of four cubs. An unfortunate day dawns in his life when he loses his tigress and the cubs due to the cruelty of human beings.

When Raja strays into human habitation, Captain (Owner of the Grand Malgudi

Circus) captures him. Holding a long whip in his hand, the Captain tames and trains Raja for his circus. Raja learns his lessons well and becomes the star attraction of the Malgudi Circus. Captain, gets overcome by greed, and allows a film to be made on Raja—who is not used to the gimmicks of the cinema world. In order to satisfy the whims and caprices of Madan, the film-maker, Captain uses a dreadful weapon—an electric metal gadget—and tries to subdue him into performing a special act, that is, to stand on his hind legs, for a film shoot. While trying to knock the dreadful weapon out of Captain’s hand, Raja, inadvertently, tears off Captain’s head, walks away from the place and enters a school.

For many days, Captain makes tiger suffer loneliness, immobility, and hunger, perhaps to break his spirit. When he loses all his strength, his cage is moved into a large enclosure and he is set free from the cage. He sees Captain standing there with a long whip in one hand and a chair in the other. He uses the whip to lash the tiger’s face repeatedly, not letting him lie down to rest, and uses the chair as a shield between them. He pokes the tiger with the chair and commands him to “Run, run, come on!”. The tiger feels dismayed and ashamed as this event is being watched by other animals, most of whom he has seen for the first time. There is a camel, a hippo, a horse, a donkey, but no deer who perhaps escaped being in this cursed place because of its past good karmas. He can recognize only a chimp. An animal advises him to perform as demanded by the ringmaster, and in return he would get something to eat and drink. The tiger asks them why they tolerate their ring-master when any one of them could stamp him out easily, and is told that once they did try it but Captain proved too strong for them. Fortunately, this conversation is carried on in animal language in the form of grunts, hissings or sighing which the “foolish” humans interpreted only as being signs of the animals’ illness. The chimp, who is the happiest animal in the circus and goes around chattering, grinning and behaving like human beings, also advises him to obey Captain and run round and round for the time being and wait for the day when they all would wield the whip and the ring-master would do the running. But for now, Captain should be obeyed so that he would continue to feed and protect them and spare them the trouble of seeking their food and fending off their enemies in the forests. According to the chimp, Captain is a fool but thinks of himself as the Lord of the Universe.

Captain’s continuous lashings teach tiger to run round and round, without there being a reason for running, after which he is put back in the cage and given pieces of meat and a trough of water. Thereafter, he is made to learn all sorts of new tricks—he is made to jump over obstacles of all kinds put in his way; he is forced to jump through a ring of fire although he dreads the fire after his past experience when he was nearly roasted by the flaming torches of the villagers; he is made to sit on a stool and sip milk from a saucer placed on a table before him, although the taste of milk nauseates him as he is basically a meat-eater and not a milk-drinker and would gladly have devoured the cow than drink its milk, as he confides to the chimp. Every time the tiger fails to perform a new trick, he is mercilessly lashed with the whip by Captain. As a punishment, he is isolated from other animals and starved for days together till he grows weak and is ready to obey his ring-master. Sometimes he thinks of attacking Captain but the Chair always shields the man. The tiger gradually becomes an established member of the circus and is not isolated any more. From the animal gossip, especially from the chimp, he gathers a good deal about their ring-master who successfully manages a vast army of people working for him—trainers of different animals, workers managing stage properties, trapeze artistes, clowns and many more. Looking after all of them, feeding them, creating new acts and tricks for the circus artists and animals, appearing on stage and looking after the finances requires a tremendous amount of energy, power and creativeness, all of which Captain possesses in abundance and which the tiger appreciates much later in retrospect.

British imperialism started by concentrating on trade. It had a policy of noninterference with the religious and cultural traditions of the people it conquered. Conquest itself was not the aim to begin with but was almost thrust upon the East India Company in its fight to protect its trade interests. The volatile political situation after the fall of the Mughal empire gave John Company (as the East India Company was popularly known) a unique opportunity to meddle in the affairs of the warring Indian princes. The Company used its leverage as a seemingly neutral outsider to its advantage. After its trading settlements were attacked, it began to fortify them and to arm itself. It raised an army mostly by recruiting local mercenaries and training them in modern, methods of warfare. In the Battle of Plassey in 1757 a small but well trained army of Indians, defeated the huge but divided army of Siraj-ud-Daula. The Battle of Plassey inaugurated a series of military victories for the British, culminating in an almost unprecedented paramountcy over the whole of the Indian sub-continent. It was through this conquest that India bore the full brunt of Western or, more properly, modern culture. This impact was extensive and thorough going so as to entirely transform Indian society. Such an upheaval, perhaps, had no parallel in Indian history. Even the impact of Muslim rule in India had arguably been less far-reaching. It is not for us to analyse or describe this impact in great detail. That would not only be outside the scope of such a course, but also somewhat tangential to our central concern, which is with Indian English poetry. It is only important to bear in mind that the British rule in India was not only oppressive, but also highly exploitative. It was a system in which India’s wealth was systematically extracted and expropriated by Britain.

Though Ezekiel is a poet of the city, in this poem he gives a living truthful rural picture. The scene of a mother stung by a scorpion on a rainy night in the village brings in its wake the two worlds of superstition and scientific temperament into focus. The neighbours swarming like flies and trying to mitigate her pain by various methods reveal the essence of community life. The father embodies the skeptic, rational approach. A telling effect is achieved in the last lines when the mother heaves a sigh of relief on her children being spared. The experience is distinctly Indian and the imagery vivid and sensitive. The neighbours concern for a speedy recovery is expressed through lines that are incantatory in effect. ‘Night of the Scorpion’ evokes superstitious practices we haven’t still outgrown. It enacts an impressive ritual in which the mother’s reaction, towards the end, to her own suffering ironically cancels out earlier responses, both primitive and sophisticated. The interrelationship between the domestic tragedy and the surrounding community is unobtrusively established. The poem also demonstrates the effective use of parallelism.

In his poem Ezekiel has done a tremendous job in depicting the Indian milieu. ‘Night of the Scorpion’ involves one entire community in a case of scorpion biting. The mother is senseless. A big preparation goes on. Each one in his manner prepares to cure “the Evil one”. The methods of superstitious practices are the main aim of the poet. But the poet never forgets to describe the plight of the victim. “My mother twisted……” However the mother’s statement cannot be overlooked. Being a typically Indian mother she wishes all kinds of problems off from her children. “Thank God….children”.
