# Supplementary material for: Dataset for classifying English words into difficulty levels by undergraduate and postgraduate students
Source: Data Brief. 2023 Oct 31;51:109744. doi: 10.1016/j.dib.2023.109744 (PMC10661753; doi:10.1016/j.dib.2023.109744)
Supplement: Supplementary file 2 [file mmc2.docx]

Ann is the daughter of Steve Deever, business partner of Joe Keller who owns a factory manufacturing cylinder heads. An urgent contract comes from the army to supply cylinder heads for aircrafts to be used in war.

But it so happens that the whole batch of cylinder heads, produced by the manufacturing firm has developed cracks. Keller calls up Steve Deever asking him to weld the cracks on the cylinder heads and ship them off to the army. The damaged cylinder heads were passed by the factory and shipped out to the army resulting in the death of twenty one pilots. There was a court case against both Joe Keller and Steve Deever. However, during the trial Joe Keller denied his responsibility for the damaged cylinder heads. The court acquitted him while Steve Deever was sent to jail where he is at the time the play opens. Frank Lubey, one of Keller’s neighbours, wants to know about Ann’s father and enquires about his release on parole. Ann wants to avoid such a question, since she is critical of her father after he was found guilty of fraud. Ann recollects that the neighbourhood had described her father and her family members as murderers after her father was found guilty of causing the death of several aircraft pilots in the war on account of defective cylinder heads supplied by him to the army. Even though Keller was acquitted by a higher court, the people of his locality still believed that Keller had got himself acquitted through underhand means.

George then accuses the Kellers to have taken away everything belonging to the Deevers. He says that he will not allow Chris to marry Ann and asks Ann to leave the place with him. After listening to George, Chris confronts his father to know whether he is the culprit. To justify his actions, Keller says that there were a hundred and twenty defective cylinder heads in the factory which he could not discard or he would have got bankrupt. He did not disclose to the army officials that he had in the store damaged cylinder heads. This would make him lose the contract and his business that had taken forty years to build. To avoid such a situation he supplied the defective cylinder heads to the army, confident that army officials would check the engines before installing them to the aircraft. Keller further says that he was sure that the authorities would send him a report after checking the engines, By the time he decided to inform them about the cracks in the cylinder heads the damage had already been done. The newspaper headlines read that twenty-one aeroplanes had crashed and the pilots had been killed. The army officials came to his factory to arrest him and he denied the charges keeping in mind his son Chris’ future. Keller says if he had let his business to collapse, he would not have been in a position to set up another business at the age of sixty one. Chris gets furious at this. He accuses Keller of killing his own countrymen. He was worse than an animal, ‘no animal kills his own’

The policeman on the beat moved up the avenue impressively. The impressiveness was habitual and not for show, for spectators were few. The time was barely 10 o’clock at night, but chilly gusts of wind with a taste of rain in them and well nigh developed the streets. Trying doors as he went, twirling his club with many intricate and artful Movements, turning now and then to cast his watchful eye the pacific through fare, the officer, with his stalwart form and slight swagger, made a fine picture of a guardian of the peace. The vicinity was one that kept early hours. Now and then you might see the lights of cigar stores or of an all-night lunch counter; but the majority of the doors belonged to business places that had long since been closed.

When about midway of a certain block the policeman suddenly slowed his walk. In the doorway of a darkened hardware store a man leaned, with an unlighted cigar in his mouth. As the policeman walked up to him the man spoke up quickly. It’s all right, officer, he said, reassuringly, ‘I’ m just waiting for a friend: It’s an appointment made twenty years ago. Sounds a little funny to you, doesn’t it? Well, I’ll explain if you’d like to make certain it’s all straight. About that long ago there used to be a restaurant where this store stands ‘Big Joe’ Brady’s restaurant’, ‘until five years ago,’ said the policeman. ‘It was tom down then. The man in the doorway struck a match and lit his cigar. The light showed a pale, square-jawed face with keen eyes, and a little white scar near his right eyebrow. His scarf pin was a large .diamond oddly set.

Jimmy Wells and Bob were both brought up in New York and were close friends. One day Bob decided to go to the West to make a fortune. The previous day the two friends--Jimmy and Bob-met at 10 O’clock at night in a restaurant called “Big Joe” and decided that they would meet at the same place exactly twenty years from that day and time-- no matter what their conditions might be or from where they might have to come.

The story begins when Bob-after twenty years came to the spot where “Big Joe” used to be and waited anxiously for his friend, Jim. A policeman on his beat met Bob there and was told by the latter about the appointment made twenty years before. While talking to the policeman, Bob mentioned that he had become rich in the West and he wondered whether Jimmy did half as well. The policeman proceeded on his beat. After Jimmy had waited for another twenty minutes for his friend, a tall man in an overcoat came directly to Bob and told him that he was Jimmy. The two men started walking up the street. When they reached a drug store, Bob-in the glare of electric lights-found that the other person was not Jimmy. The story reaches a climax or a point of revelation when the other person revealed his identity as a policeman and informed Bob that he was there to arrest Bob, the gangster-who was wanted by the Chicago police. This revelation is followed by another revelation when Bob, from the little piece of paper handed over to him, comes to know that the patrolman whom he has met at the appointed place was Jimmy, who has came to keep the appointment.

At the beginning of the story, the author introduces Jimmy Wells: He is a policeman with an impressive walk. He has ‘stalwart form and slight swagger’ and he looks like the picture of a guardian of the peace. It is, indeed, the description of conscientious and impressive policeman. After giving the above description of Jimmy Wells, the author tells us more about him through the words of another important character, Bob. According to Bob, Jimmy is “the finest chap in the world” and is ‘always the truest staunchest chap in the world’. The same technique-the technique of delineating a character through description, dialogue, and incident-is employed by the author with regard to Bob as well. When Bob lit his cigar, ‘the light showed a pale, square-jawed face with keen eyes, and a little white scar near his right eyebrow’. His scarfpin was large diamond, oddly set, indicating his acquired affluence. Through Bob’s words about himself, we come to know more about him: He competed with some of the sharpest wits in the west and became rich. Even though he is successful in the West, Bob never forgets his friend, Jimmy and the appointment with him. We see the positive side of his character-his belief in friendship and his affection for his friend-when he comes after twenty years to the appointed place. As we know, every person has several traits-positive as well as negative. Along with Bob’s loyalty as a friend we Also find about his criminal record in the west, when the person in the long overcoat arrests him. Having discussed the two important characters, we shall now proceed to a discussion on background and atmosphere.

The poet cannot remember where the thought went to or why such a thought occurred to her. That is, the occasion of the thought or the source which inspired it is also kept in the dark. Why it recurred too, is unknown. The poet says in clear terms that she cannot say definitely what it was, thus emphasizing its shapeless and elusive nature. The poet says that she lacks the skill to describe it in specific clear cut terms. Deep within her soul she knows that it is familiar to her. That is why she is able to realise that it has occurred to her in the past. The reappearance of the thought was just a reminder and it never comes to the poet again.

The poet is unable to give a precise expression to the thought. But a thought exists when we can say what it is. An author can express any thought that crosses her mind, at least approximately. The poet insists on keeping us in the dark about the nature and reason behind the thought. Therefore, it is obvious that thought itself does not form the subject matter of the poem. The thought becomes a metaphor for the mysteries of human existence. It may allude to life itself which we cannot define in precise terms in spite of the fact that we have awareness about it. Again, the inexpressibility of the thought may also refer to the mysterious working of the human mind. The working of the mind is as unpredictable as the appearance and vanishing of the thought and is as indescribable as the elusive thought that defines explanation in specific terms. It may also refer to the creation of art. A work of art emanates from the creative mind of an artist but how it evolves or why it came into being remains a mystery.

It is a well-known fact that the Native American people had their land taken away from them by the European settlers that came here in the 17th century. It's a tale that many children hear in their history classes. But what many of them don't know (and what the American government is not teaching) is the way in which their lands have been taken away. There is no doubt that there have been some violent struggles for land between Native Americans and the European settlers but what is not shown is how mainly trickery (not violence) was used to steal their lands from them. The main trick that was used to get Native Americans to sign off their lands to the Europeans was the act of introducing alcohol. The Europeans knew about alcohol's ability to create addiction, intoxication, and impair a person's mind. So they used this as a tool to take away the valuables that Native Americans owned. Alexie illustrates this point when he writes the story of how "Buffalo Bill opens up a pawn shop on the reservation right across the border from the liquor store". Buffalo Bill knows that the Native American can't resist alcohol and systematically sets up a shop right across a liquor store so that the Native American people will feel tempted to go to the liquor stores after pawning their "jewelry, Television sets, VCR[s], and beaded buckskin outfits." He manages to make money from them "taking everything [they] have to offer" but we don't see how the Native American people benefit from this. Another thing worth noting is that Buffalo Bill "stays open 24 hours a day, 7 days a week". Alexie is showing how those who are plotting against the Native Americans don't take a break, they seek to gain profit in any way they could at any time of the day.

There are various ways of looking at this category of writings that comes under the broad umbrella of Children’s Literature. However, we could define it broadly as writing/ visual narratives that have been written, designed and developed to entertain and largely instruct youngsters. Children’s Literature would therefore encompass a wide range of works such as the good old classics; works from across the globe; pictorial story books, comics, graphic novels/ narratives, fables, folktales, fairy tales, lullabies, nursery rhymes, as well as orally narrated folk tales, folk songs and legends. Some scholars define Children’s Literature as books written for children, some leave out comics, joke books, cartoon illustrated books, encyclopedias for children and other non-fiction such as autobiographies/ biographies. However, it may be useful for us to take into account that we as students of Popular Literature, need to realise that the genre of Children’s Literature is broad and inclusive with blurry boundaries. We might be aware that traditionally, Children’s Literature as stated earlier, was aimed at educating and orienting children to adult expectations by imparting lessons in social propriety and inculcating the right moral values. For instance, Victorian society tried hard to influence its children through the medium of stories that were moralistic in nature and didactic at heart. Children’s Literature in a sense used and uses make-believe, fantasy as opposed to reality. Children’s Literature began with oral story telling being passed down from one generation to another. It also needs to be remembered that for the longest time, children were not thought to be distinct from adults, they were considered to be miniature adults during the Puritan Age.

It is with the birth of the representation of literature on celluloid that a unique relationship between literature and cinema was established and its strong bond is visible even today. These critics were concerned with the fact that the common man was mixing up real life with reel life. Cinema, as one of the dominant technologically progressive modes of cultural transmission has positioned itself as integral to the development of the ‘cult of the pop’ in modern societies. As mass public culture, art practice, vehicle of propaganda and adaptations, cinema creates an extended narrative text for literature. Thus, questions like — what happens to literature in cinema and can pedagogy remain unaffected by its representations in cinema gains greater relevance now. In the last two decades, a paradigm shift has taken place, whereby a text has been placed against various production apparatus — cinema, stage, television, comic books, etc. If literature has to fulfill its roles and purposes and be a communicative practice, the analysis of popular narrative (fiction) can provide a crucial link between the literature and study of other modes of representations. In the next section we shall examine popular Indian English Fiction. The Indian English popular fiction, rooted in the concerns of the present, voicing the aspirations of the young and new Indian middle class, is redefining the canon. Unknowingly, in its bid to respond to the cultural effects of consumerism, moral anxieties over shifting gender roles and changes in youth culture, and its drive to become self-consciously less literary, it is playing a pivotal role in canon formation.

The book opens with Alice mock scolding her black kitten for unwinding a ball of yarn. While the boys are out in the snow gathering sticks for the bonfire, *Alice* being a girl is confined to the house and can only amuse herself through ‘pretend’ games. *Alice* is presented as an imaginative child with a propensity to daydream as she lists the mistakes Kitty has made, all along talking to herself and pretending she is in a dialogue with Kitty. Playing with chess pieces, *Alice* asks Kitty if it plays chess and then putting the Red Queen piece before Kitty asks it to imitate it. Scolding Kitty in the manner of a Victorian governess, she threatens to put Kitty into the looking glass house for the mischief. We see *Alice* thinking about the chess game as she falls asleep and enters the fantastic world of the looking glass. The story runs like the dream of a half-asleep child in which *Alice* magically crosses over to the other side of the mirror into the world of looking glass. The use of fantasy not only establishes daydreaming as a motif in the text but also presents the restricted life of young Victorian girls who were denied the freedom available to boys. The entire book is structured in the form of a chess game. Conventionally, chess is taken to be a game for the adults since it involves certain well-defined rules and thought-out irreversible moves. The use of chess as a motif is also reminiscent of the fact that Carroll taught chess to the Liddell sisters and even invented the traveler’s chess. The chess motif that runs throughout the text becomes a key to the narrative. The game represents a map for the entire book and can be read at multiple levels: at the physical level, at the metaphysical level, and, at the dream world level of the looking glass.
