# Supplementary material for: Dataset for classifying English words into difficulty levels by undergraduate and postgraduate students
Source: Data Brief. 2023 Oct 31;51:109744. doi: 10.1016/j.dib.2023.109744 (PMC10661753; doi:10.1016/j.dib.2023.109744)
Supplement: Supplementary file 3 [file mmc3.docx]

Robinson Crusoe is not an epic hero who goes on fantastical adventures, but a protagonist of the text which has been hailed by posterity as one of the first English novels that deploys a "realist" mode to tell a story about a character's extraordinary but believable bildungsroman. The narrative introduces Crusoe as a son of a merchant who leads a relatively genteel life now and desires to bequeath his wealth to Crusoe. Despite his father's repeated counselling, Crusoe is unable to suppress his longing to go on sea voyages and finally gives in to temptation. His rejection of the paternal inheritance and authority are symptomatic of his breaking away from established traditions and going on a quest for re-making his life. The narrative as a whole bears testimony to Crusoe's ingenuity and resourcefulness. These traits become all the more pronounced during his sojourn on the island where he not only survives for so many years but also toils hard to carve a comfortable retreat for himself. He perseveres for months to build a canoe, and relentlessly practices pottery making till he masters the art of it. He builds a home, a shelter for his goats, a country home, a grapes arbor, a dairy, all from scratch and keeps a journal so as to maintain a sense of order. These are crucial moments in English literary aesthetics as Crusoe's assertion of will and gentility signal a new kind of hero - someone who is not living in an absolutely pre-determined world but is a self-realised individual. The Genevan philosopher Jean-Jacques Rousseau's high recommendation of Crusoe's hands-on approach in his fictional text, Emile, also plays a major role in making Crusoe's experiential learning proverbial.

Despite all his faults and abhorrence of human beings, Gulliver's ability to empathise with the inhabitants of the new islands he claims to have discovered, makes him a hero. No matter what, he wants to protect them from British colonialism. Gulliver, in the end, turns out to be morally superior to both Englishmen and several other characters he encounters during his voyages. His moral pre eminence stems from his ability to respect the bond between a host and a guest. Lilliputians do not respect the bonds of hospitality. They conspire against their guest who protects them from the neighbouring enemy and, therefore, they are morally depraved. In Book I, while they were walking all over Gulliver's body, he was tempted to seize forty-fifty of them and dash them against the ground. But Gulliver resists this temptation, as he feels bound by the "Laws of Hospitality to a People who had treated me with so much Expense and Magnificence". Unlike the Lilliputians, the Houyhnhnms treat their guests hospitably. They value friendship and benevolence. They treat a stranger from the remotest part of the island as affectionately as they will treat their nearest neighbour. Thus, both Gulliver and the Houyhnhnms, present us with the model of a good guest and a good host respectively. Gulliver continues playing the role of a good host and does not betray anyone after he leaves the islands. The safety of the inhabitants of these islands is his prime concern. He breaks the law of his country but not the trust of his friends. Hospitality was a recurring trope both in the classical texts of Greco-Roman culture and in Gulliver's Travels. In the Homeric world, like the world of ancient India, it was a sacred bond. Both the Iliad and the Odyssey are full of references to the warm welcome extended to guests, including strangers by the host.

Dickens wrote during the Victorian age, when Queen Victoria was ruler of England. It was above all the age of great social change, marked by the dates of the two Reform Bills of 1832 and 1867 —the period of Dickens' literary production as well. The Reform Bills gave franchise to working-class men in response to movements like Chartism, which made demands for greater democratic participation in the government. A number of other legislations were enacted in such areas as factory reform, wages, education, public health, divorce and inheritance for women, trade and agriculture. The success in achieving reform measures in England was directly related to revolutionary movements in the rest of Europe, particularly France. Dickens was an active campaigner for reform, arguing, like many other Victorian thinkers, that this was the only way of staving off violent social upheavals like revolutions. This connection will be discussed in greater detail in the next units, in the specific context of A Tale of Two Cities. The Victorian age - especially after the 1850s - was also an age of progress: it was a period of rapid industrialization, imperial expansion and population increase, all of which led to overall material prosperity. The resulting feeling of nationalist pride could often sound complacent and jingoistic. Therefore, many of the writers of the time, It Carlyle, Ruskin, Dickens and Morris directed their social criticism towards the materialism, the continuing economic and social disparities, the philistinism and the aggressive temper of the age, though at the same time these writers often shared the contemporary belief in progress.

The French revolution was a protracted and complex phenomenon and not a spontaneous cataclysm that Carlyle and other British writers made it out to be. The constant comparisons between the French revolution and a tempest or an earthquake, however, were not innocent, since these implied that the events in France defied all forms of understanding. Moreover, writers as diverse as Burke and Carlyle never hesitated to use metaphors and images and heightened language to subsume the facts and the achievements of the French revolution in an almost mythological sense of horror. Dickens' A Tale of Two Cities comes basically out of this matrix of attitudes, but we hope you have noted how Dickens both draws on but also departs from the writings that taken together constitute the conservative English response to the French revolution. We have seen in what respects Dickens' treatment of the French revolution differs from that of Burke and Carlyle. We have seen that while Dickens holds the aristocracy responsible for precipitating the revolution, he is not sympathetic to the revolutionaries either. He depicts them in diabolical term, associating them with indiscriminate bloodshed and vengeance. Dickens' treatment of Mme. Defarge, however, is more complex as he treats her with fear and hatred as well as with admiration. Dickens' views differ from Carlyle regarding the revolution and the situation in England. Carlyle draws parallels between the situation of France in the 1790s and the England of the 1830s. Dickens does not feel that the events of France could be repeated in England. Carlyle talks about the oppression and decadence of the aristocrats and feels that they were responsible for precipitating the revolution.

I remember a touch of conscience in this kind at school. My good old aunt, who never parted from me at the end of a holiday without stuffing a sweet-meat, or some nice thing, into my pocket, had dismissed me one evening with a smoking plum-cake, fresh from the oven. In my way to school (it was over London bridge) a grey-headed old beggar saluted me (I have no doubt at this time of day that he was a counterfeit). I had no pence to console him with, and in the vanity of self-denial, and the very coxcombry of charity, school-boy-like, I made him a present of -- the whole cake! I walked on a little, buoyed up, as one is on such occasions, with a sweet soothing of self satisfaction; but before I had got to the end of the bridge, my better feelings returned, and I burst into tears, thinking how ungrateful I had been to my good aunt, to go and give her good gift away to a stranger, that I had never seen before, and who might be a bad man for aught I knew; and then I thought of the pleasure my aunt would be taking in thinking that I -- I myself, and not another -- would eat her nice cake -- and what should I say to her the next time I saw her -- how naughty I was to part with her pretty present -- and the odour of that spicy cake came back upon my recollection, and the pleasure and the curiosity I had taken in seeing her make it, and her joy when she sent it to the oven, and how disappointed she would feel that I had never had a bit of it in my mouth at last -- and I blamed my impertinent spirit of alms-giving, and out-of-place hypocrisy of goodness, and above all I wished never to see the face again of that insidious, good-for-nothing, old grey impostor.

As her early years went on with the absence of formal education she faced the challenges of learning from home because of her stepmother. On the other hand, her pre adolescence was even more challenging and compelled her to learn more and more inspite of all the hurdles. Her family dynamics soon turned even worse at the beginning of her pre adolescence. As mentioned above, Godwin’s business was not improving and did not see a sufficient amount of profit. Due to these critical circumstances, he was forced to borrow substantial sums to run the family as well as his business from new lenders to pay off the earlier loans. Later, when his business was close to failure and he was pushed to the edge of despair, Godwin was saved from debtor’s prison by his philosophical devotee who helped him with more money. Mary Shelley was used to this kind of family dynamics during her teens. She learned to deal with all these sorts of disturbances by taking her refuge in writing and often spent her time dwelling in imagination. She grew up as a bibliophile. As we know, her father tutored her in a range of subjects and she started using her father’s library. She felt that the library was the only place of escape from the hustle and bustle of her life. Godwin often took the children on educational outings through which Mary learned many things. She used every opportunity to the fullest that was offered to her and felt that even if it is a small set of circumstances it may help her with a big basket. Although Mary Shelley received formal schooling, yet the primary mode of her education was largely unconventional. Her father supervised her education and she was tutored rigorously in various subjects including French and Latin. Her rich and eclectic education further evolved through an interaction with her father’s contemporaries.

The story begins with a captain Walton taking his ship into the Arctic Ocean with his crew. He was an English adventurer who was hoping to make some important scientific discoveries by undertaking a voyage in a ship as a part of exploring the North Pole. Due to severe winter, a sheet of ice formed all around the ship and so it got stranded for a few days. It was damn cold that day and Captain Walton and his crew were exploring various possibilities to escape from that icy spot. Suddenly, they heard some strange sound from the west side. Walton, through his telescope, saw an eight feet tall gigantic man who was driving a dog-sled across the ice. After a while, he along with his crew witnessed another man of average size on another dog-sled chasing and trying to shoot the first one, but he stumbled and slipped down from the sled. Walton asked his crew to rescue the man from the ice where he was almost dead from exhaustion and exposure to freezing cold wind. As an eleventh hour Samaritan, the captain decided to take him aboard with them. They treated him and helped him recover from the trauma. The man was none other than Victor Frankenstein. While he was recovering, Walton started making enquiries to Victor about his identity and that gigantic man who was chasing him. Thus Victor narrated the story of his life or the origin of the monster to Walter which was narrated by the captain to his sister in the form of epistles. Since childhood, Victor had his interests in scientific experiments and an unquenchable thirst to accomplish something new in the field of human science and anatomy. He was also obsessed with studying outdated theories that focused on simulating natural wonders. Victor had his friend, who was always hanging out with him. His mother Caroline died of scarlet fever a few Weeks before he was planning to go to the University.

At the university, Victor mastered the subject of chemistry and excelled in other sciences which propelled him to develop a secret technique to impart life to non-living matter. At the end of the first day’s class, Victor met his professor Mr. Waldman and discussed about his interest in scientific experiments and his ideas on creating life from the dead. The professor could see a spark in him and his aspiration in making some innovative experiments using new ideas in the field of human science. Hence he helped Victor in the initial stage of his series of experiments by teaching him about galvanism. In medicine, galvanism refers to any form of medical treatment involving the application of pulses of electric current to body tissues provoking the contraction of muscles that are stimulated by the electric current. Victor tried applying his ideas using galvanism on a dead frog and as a result of the experiment, the legs of the frog twitched upwards. This incident gave him a sudden motivation and encouraged him to indulge in wilder imagination. He thought that if he could stimulate the frog then why couldn’t he produce the same reaction from the heart and muscle of the human corpse? Victor told his ideas to his professor but the professor warned him not to try any unorthodox scientific experiments infringing nature’s law. However, Victor was never to give up his enthusiasm on this idea of resurrecting life from death. Eventually, he embarked on the process of creating a humanoid. In order to carry out this experiment, he exhumed different body parts of corpses from the graveyard. Due to the difficulty in replicating the minute parts of the human body, Victor made the Creature tall: about 8 feet in height and proportionally large. Despite Victor’s selecting its features as beautiful, upon animation the creature was hideous with watery white eyes.

There is a possibility and a dire need of drawing a parallel between Victor and the monster. Victor in the novel is shown as a man of average virtues who is very much connected with his family but not with the outer world in a great deal. On the other hand, the monster is left in complete isolation. He was not gifted with a family or a friendly circle with the rest of the world. His wretched appearance had branded him as ‘‘anti-social entity’’ which provided a license for the society including the creator to isolate him and treat him as fiend. The irony lies in Victor’s unwillingness to comprehend the problems of the monster from the monster’s perspective. This injustice rendered to the creature has constructed the antisocial self of the monster for which Victor is morally responsible for creating a situation where the monster is pushed to the extent of committing a series of murders only from Victor’s family. David Punter, in the same article emphasizes on the repercussions of such injustices rendered to anyone including the monster in the following lines. “A great deal of gothic is about injustice, whether it be divinely inspired, or meted out by man to his fellow men and women. On the whole, the novel is a kaleidoscopic representation of various scientific, anthropological, and sociological issues which are still baffling the humanity. Victor is the protagonist of the story and a scientist who creates the monster. His diligence and credence on his unorthodox scientific experiments eventually led to his own ruin. He found a mission from the ideas of bringing back the dead to life and so he devoted his life to experimenting in the same. He was supposed to take care of the creature which was created by him, but his irresponsible behaviour brings tragedy to his life. He took no care in protecting his family and his friend Henry.

‘Pied Beauty’ which is one of Hopkins’ happy poems, is a hymn of creation that praises the creator by praising the created world. It glorifies all the things on this earth that are either ‘pied’ or spotted. The poet thinks that it is a manifestation of God’s creativity. With the eye of a painter, Hopkins vividly sketches in kaleidoscopic variety, all the objects and patterns which provide an example of this kind of beauty. Hopkins starts with a eulogy of Lord the creator: “Glory be to God for dappled things”. This is followed by an inventory of things which are dappled or spotted. He includes in this list, the sky that is dappled at dawn, with blotches of blue colour splashed against pale white, the contrast described as ‘couple-colour’ by Hopkins. It reminds him of “brinded cow” or ‘brindled’ or ‘piebald’ cow, whose hide is again a contrast of brown against white. Then he describes the trout fish which swims, that has its body painted [stippled] with rose coloured moles. The next image, a complex one, is of a chestnut, the meaty interior cradled within its hard shell falling out, hiding its smouldering brilliance like coals in a fire, black on the outside, but glowing within as it splits and falls. The tiny birds, finches, are multicoloured usually with specks on their wings; and the landscape of a farmland, enclosed in patches, forms a pattern according to the way in which it is cultivated or left fallow or freshly ploughed. The last example in the octave is taken from the world of man, where the tools and equipments of his trade, make a dappled pattern in their variety. Hopkins places man in his context – he is only a part of the extensive natural world. And even human achievements such as trade, gear, tackle and trim, can be seen only as a part of the larger scheme of things.
