# Supplementary material for: Dataset for classifying English words into difficulty levels by undergraduate and postgraduate students
Source: Data Brief. 2023 Oct 31;51:109744. doi: 10.1016/j.dib.2023.109744 (PMC10661753; doi:10.1016/j.dib.2023.109744)
Supplement: Supplementary file 4 [file mmc4.docx]

The Victorian Age in England, especially the second half of the 19th century saw rapid growth of industrialization and remarkable advances in the sciences and the social sciences. Industrial progress resulted in a huge exodus from the rural areas to the cities that brought about distinct social changes. In social sciences such as psychology, Freud's theories explained human behaviour and introduced psychoanalysis for treatment of mental illness. New philosophical inquiries and political theories needed alternative modes of expression.

Modernists felt a growing alienation from Victorian prudery rooted in Victorian morality and in a. society based on hierarchical principles of gender and class, its optimism, and conventions. New ideas in psychology, philosophy and political theory, kindled a search for new modes of expression. All these caused a radical shift both in form and content in 20th century art and literature.

Modernism introduced new literature and new forms of art that were innovative and experimental. The first three decades of the 20th century (approximately 1900-1930) is called the modern period. The postmodern period started around the time World War II ended, approximately after 1945. It spanned the second half of the 20th Century almost for three decades and gained ascendancy over modernism from the 1960s. However, we have to remember that artistic and literary movements cannot be pinned down to an identifiable calendar year, and we cannot say that modernism started exactly in 1900 at the turn of the millennium, and postmodernism at the end of World War II in 1945.

Broadly speaking, the 20th century saw the rise of a new movement in creative arts, both visual and performing arts. It had a strong impact on literature, theatre, painting, sculpture, music, dance and architecture. It was also a philosophical movement as it effected a change in the Western society towards a new way of thinking, living, expressing and engaging in cultural and artistic pursuits. Modernism as a movement gave men and women the means to tackle a new world that was increasingly getting impatient with traditional mores and beliefs. The term Modernism has come to signify a new trend in the early decades of the 20th century, a divergence from the earlier tradition of arts and literature that brought in various innovative movements and styles as a replacement. Among the many factors that gave rise to the new movements – modernism and post modernism - two are significant: (1) the rise of the new modern industrial societies that contributed to the rapid growth of cities and (2) the two horrific World Wars which resulted in great destruction and extensive loss of life, in particular, the racist genocide on a vast scale in the name of ethnic cleansing. The kind of inhumanity evidenced in the two World Wars was not a new phenomenon. Violence and cruelty between man and man, united by common ancestry, but divided by race, religion and society, have always been a part of our existence, from the days of *the Mahabharata* War. Modernism, true to its name represented a clash between the old and the new. It rejected all traditional forms as irrelevant and outdated to fit in with the new economic, social, and political environment of a modern competitive, industrialized world.

This also leads to changes in the spatial lay-out of the town when the ‘thatched bulging cottages’ are replaced by ‘great quadrangles of dwellings’. The geographical survey and historical perspective that the Barons mention are effected in this part of the novel by the way it records changes in the cultural landscape of the region, and in the change of the place through time. Throughout the opening paragraphs the sense of change in the cultural landscape is constantly built up. The transition that so radically altered the cultural landscape of Bestwood is built up through a series of contrasts. Hell Row with its thatched bulging cottages clearly contrasts with the Bottoms and its great quadrangles of dwellings; the little gin-pits are contrasted with the new mines; the gin-pits were worked with donkeys, while the new mines depend on a fairly extensive system of railways. There is the implication that mining on a small scale had been a traditional economic activity in the area for years without interfering in the local ecosystem (hence the reference to the brook scarcely soiled by the little gin-pits) and generally blending into the rural landscape, merely forming ‘queer mounds’ and ‘little black places’ (*Sons and Lovers*: 9). The “sudden change” has led to the little gin-pits being elbowed aside by the large mines, completely altering the landscape which had known hardly any change for years. The railway runs across the wooded countryside and the cornfields, sharply emphasising the contrast between the old and the new. If the miners who earlier worked the little gin-pits lived in little blocks of cottages scattered here and there in the village, the regiments of miners who work in the new mines are housed in the huge blocks of standardised company housing.

The focus on the cultural landscape, as altered by the operations of the mining company in the early sections of the novel, foregrounds the significance of mining in making Bestwood what it was at a particular point of time in the history of the region. Another local occupation which figures in the novel is the hosiery trade. The women in the Bottoms are shown waiting for ‘Hose’ the hosiery agent. Hose distributes machine knitted pieces of stockings among the local women who stitch the pieces together and return them to him. It is work that demands time and skill and is not well paid, but several women of the Bottoms are shown turning up with their work, apparently glad to make the extra money and to augment the income of their miner husbands. The exploitative nature of the process is hinted at throughout the passage. Mrs. Morel, unlike her neighbours, refuses to do such badly paid work and makes her disgust with the work and the hosiery agent quite clear. Arthur Coleman explains how framework knitting at one time equaled and surpassed agriculture as the basic form of employment in Eastwood, but started to decline from around 1800 due to the newly developed coal mines which offered more lucrative and more secure work. By the time we come to the historic period framed in the novel, framework knitting had ceased to be primarily a cottage industry. The industry had been mechanised and only a minor job was to be done by hand, and it is this part which is performed by the women outworkers.

There is a justification in saying that the peculiar manifestation of Modernism was the Stream of Consciousness emerging in a new form of fiction. It gave literary writing a peculiar character adhering to the logic of spontaneity in thought. That indeed turned the table on how thought had been considered so far. At the turn of the century, a belittling of thought occurred. The presence of logic was rejected in representation. That undermined the nature of thought steadying the boat of life, so to say, and leaving the mental process to the exigencies of moods and emotions. The sense of certainty in the human mind was done away with. Thus, Modernism and the Stream of Consciousness worked hand in hand to establish the superiority of human being’s mental processes in a raw form. The paradigm appeared in the form of thoughts of the characters in works of fiction without a specific pattern. Literally, it was a flow. We are made to realise that consciously chosen endeavours of a character relate, directly or indirectly, with his/her self, thus going over innumerable matters floating in memory as thinking and memory work in tandem. In the stream of Consciousness fiction, a character was shown as waking up in the morning and soon beginning to sort out his or her dilemmas with a dreamy pressure of the previous day. The age of modernist flowering was conflict-ridden with no tangible contours. It was recognized as a fact of modern-day life and appeared to be the circumstance going out of control. That was contrary to the perceptions of Woolf’s predecessors. Gone was the time when human beings by themselves decided on a path to pursue, when a tangible aim worked out with effort at the larger cultural level and was the determining principle of their behaviour.

Mrs. Dalloway does not offer statements or descriptions. The author does not seem to be interested in telling a story. It may even be said that there is no story to tell in the novel, indeed the novel can do without it. Only in the background does Mrs. Dalloway have a narrative—a sequence of happenings in which characters participate. The individual circumstance and the social situation are suggested through the dialogue that the central character holds with herself. That too, happens in her memory. This method of representation is consciously used by the writer. We note that the writer’s choice is to focus on the circumstance of an individual who is driven by the logic of her world. We might contrast this with the traditional fiction form that, among other things, worked at the level of dialogue. In the traditional novel, people talk, discuss, disagree, use satire, or create a climate of happy exchange. All these are conspicuous by their absence in Mrs. Dalloway. The novel presents an account of a mental state, the one in which Clarissa finds herself involved. We may wonder whether such a novel would be able to engage the reader. On the other hand, Woolf would insist that her job is to explore and examine a phenomenon she confronts in life. She would take the existing circumstance as a challenge. The assumption is that the alert citizen would need to know the surroundings from an angle of interest and serious concern. If there are spots in society that present a problem, the citizen would pause and think about them. In that process, he would mentally participate to see the dynamic link between one thing and another. That is the point Mrs. Dalloway raises.

In Mrs. Dalloway, there are many characters facing grave issues. The string of their thoughts is held by an omniscient narrator. The thread connecting those characters with Clarissa is time. They coexist with Clarissa in the London of the nineteen twenties. Also, Clarissa is aware that the world she inhabits is a complex one, ridden with cross purposes. She would come across many people on the road she does not know. But they do exist. This fact might explain the fact that her life, family and neighborhood also reflect those of others outside her personal circle. The example of Septimus Warren Smith and his wife Lucrezia comes to mind. Septimus is an Englishman and his wife is an Italian. He is a soldier who fought in the First World War. There, he had a friend called Evans. The two fought together on the war front till one day; Evans got killed by a gunshot before Septimus’s eyes. Septimus has carried all along the guilt of Evans’ death. This gives him a split personality. He hallucinates many a time in the novel seeing the ghost of Evans and he enters imaginatively into the scene of the war far back in time. This gets him in conflict with the sweet and harmonious life with Lucrezia. Because of Septimus’s hallucinations, Lucrezia remains concerned all the time about the safe upkeep of her husband and follows to protect him like a shadow. The point made is that Clarissa and Septimus will never meet, yet they constitute an important part of the novel since they coexist in that world. What would it say about the novel except that the narrative in modern fiction is non-linear and integrated on the strength of distant associations?

The poem "The Second Coming," was written in 1919.Yeats wrote the poem to coincide with the end of World War I (1914-18) that had caused suffering and death, torture, hunger and disillusionment to millions of people. The First World War had let loose anarchy in the world. During the War, bombs were dropped, and many thousands of innocent people were killed which made people think that the time had come for the end of the world. The War proved catastrophic, almost signaling the end of the world and people began to wait for relief from the cruel happenings. This parallels the waiting the second coming of Jesus to earth, as revealed in the Book of Revelation (the last Book of the New Testament). In the New Testament, it is stated that the second coming would happen at the end of a catastrophic war and that the Saviour would arrive and take all devout people to a joyous life in heaven. The apocalyptic prediction of the collapse of civilization as revealed in the Book of Revelation seemed truly prophetic in the context of the deadly world War. The New Testament made many Christians long for Jesus’s return to the world a second time as their Saviour. The concept of time span is revealed by the title with the three words, ‘The Second Coming’. Just as Christ’s earlier arrival in the world was for saving men and women from their sins and punishments, in the same way the poet feels the second coming of Jesus will be to save mankind from total annihilation as evidenced in the First World War. The poem is difficult to understand with its shocking imagery and obscurity. Once the images are well explained, it is easy to apprehend the theme of the poem. Simply stated it is a lament for the death of the old world and an expression of hope and expectation of a possible rebirth of a new one.

The poem “The Unknown Citizen” is simple, direct and uncomplicated. It is easily comprehensible as the theme relates to our times where the discordance between the State and the people is a quotidian experience. The poem is in the form of a satirical elegy, where two literary genres - ‘elegy’ and ‘satire’ are fused. What is an elegy and what is a satire? An elegy is a song or poem expressing sorrow or lamentation especially for one who is dead. A satire is a literary form in which human or individual vices, follies and shortcomings are criticized by means of ridicule, derision, burlesque, irony, parody, caricature, or other methods, though at times with an intent not so much to mock but to inspire social reform. In a satirical elegy, the elegy is used to make it perform a task directly opposed to its intended purpose. The author laments the loss of man’s individuality by making him conform to the rules laid down by the government/society to ensure its smooth running. The Unknown Citizen never deviates from those norms and therefore Government has no complaints to make against him - holding him as a model of disciplined citizen, but in reality, a man's life is so much more than mere compliance to set rules. Such life negates all exercise of human powers, emotions, desires and wants. From the language and content of the poem it is possible to surmise the speaker to be a spokesman of the government, a bureaucrat who addresses the elegy to an individual referring to him not by his name but by alphabets and numbers. This form of address by the heard but unseen voice of the speaker sets the tone of the poem. It is impersonal, officious, lacking in sensitivity and empathy. The speaker represents the faceless, indifferent and ruthless bureaucrat of the State who exerts his authority on the nameless citizens masquerading as a concerned and benevolent power.

The 1930s marked a period between the two deadly World Wars. The first World War was between 1914 and 1918, the second between 1939 and 1945. The two Wars devastated Europe and brought about a change in people’s perspective on the meaning of life, their faith in God almost bordering on atheism and nihilism, the collapse of all values that had hitherto been regarded as hallowed and inviolable. The established world order had crumbled and idealists like Spender turned to Communism as an alternative to Fascism. Europe was reorganized, and a new world was born. The European nations that had fought in the Great War emerged economically and socially crippled. Economic depression prevailed in Europe for much of the inter-war period. “The political atmosphere of the inter-war years was sharply divided between those who thought the extreme left could solve Europe's problems, and those who desired leadership from the extreme right. This situation kept the governments of Britain, France, and Eastern Europe in constant turmoil, swinging wildly between one extreme and the next. Extreme viewpoints won in the form of totalitarian states in Europe during the inter-war years, and communism took hold in the Soviet Union, while fascism controlled Germany, Italy and Spain”1 . Spender went to Spain during the Spanish civil war to fight the Fascist forces under General Franco, though he personally did not participate in the fighting. Instead he wielded his pen to write Poems for Spain. From his early days Spender was concerned with social and political issues and gained celebrity status as a poet. But in his post -war life, his focus was more on prose that included literary criticism and fiction and also drama.
