# Supplementary material for: Dataset for classifying English words into difficulty levels by undergraduate and postgraduate students
Source: Data Brief. 2023 Oct 31;51:109744. doi: 10.1016/j.dib.2023.109744 (PMC10661753; doi:10.1016/j.dib.2023.109744)
Supplement: Supplementary file 5 [file mmc5.docx]

The postcolonial writers were all from among the subjugated class of natives; they were born to the colonized population, had the privilege to get educated and learn the language of the colonizer which in most of the cases was English, wrote in their language and continued to write more fearlessly and with greater authenticity even after their country got political freedom. Like Caliban, they used the language to their own advantage as a tool to expose the cunningness, meanness, greed and cruelty of the colonizers or the colonizing nation while also, at times, acknowledging the constructive modernization and advancement brought by the colonizers. There were only a few exceptions in the form of the white writers who like Sinclair, the British writer represented those who migrated to the common wealth countries to settle there permanently and wrote about the colonial experience from the point of view of the oppressed. They rejected the colonial hegemony and recognized endless possibilities in the indigenous potential and the storehouse of immense knowledge inherited and developed through generations. Some of the foremost texts which carry a discourse on racist undertones and take up the issues of political and cultural independence termed as postcolonial are mentioned here. These canonical texts paved the way for the postcolonial movement which offered to the world a body of literature diverse in content but unified and unique in its appeal. There are many other writers of repute who made valuable contribution to the corpus of postcolonial fiction. To make you aware that the postcolonial writers’ world consists of a mammoth body of literature which is still growing and to acquaint you with its pioneers and star writers, some names along with their leading works are mentioned above.

Edwidge Danticat: This Haitian American writer’s stories mainly revolve around the themes of national identity, revolutionary struggle, diasporic politics and mother-daughter relationships. She has written many novels. Her story “Ghosts” is about the life of Pascal, a slum boy whose parents used to rear pigeons for a living in their village. People would buy these pigeons for a bizarre custom which his parents found disgusting. They moved to the outskirts of a city and started a restaurant. His brother Jules moved to Canada with his girl friend while Pascal worked as a news writer for one of the most popular radio stations, Radio Zorey. Gang leaders and other criminals used to visit the restaurant and talk freely about their activities. Once when he overheard Tiye, a one armed, bald headed gang leader it struck to Pascal that he could run a radio programme “Man to Man” on these people. He discussed his plan with his friends and many gang members also came to know of Pascal’s plan. His idea was stolen by other people and they started airing the programme on Radio Zorey. Pascal was teased by the visitors and they instigated him to take action but Pascal did not want to react. Radio Zorey building was torched by the Tiye gang and Tiye named Pascal as the mastermind. Pascal was suddenly arrested and given third degree torture in the prison cell. His family including his brother spent huge sums and made all out efforts to free him but failed. When Tiye struck some deal with the police and the judges Pascal was released. Tiye visits their restaurant again and Pascal by noticing his prosthetic arm dreams of running a radio show by the name “Ghosts” on people with lost limbs.

“The Girl Who Can” is a short story written in an oral style. The very first line ‘They say that I was born in Hasodzi; and it is a very big village in the Central Region of our country, Ghana ‘sounds like an anecdote orally recounted by the young narrator. Written in first person narrative, it deals with the life of a seven years old girl, Adjoa who lives in a house with her mother whom she calls Maami and her grandmother whom she calls Nana. Adjoa is a bright young girl who finds it hard to express herself without being silenced or ridiculed. She is often perplexed at the duality of Nana’s approach. She is either met with a shocked Nana who forbids her from repeating her statements sometimes, and other times she finds Nana racked with laughter over their childlike innocence and repeats them to her friends and neighbours. Young Adjoa is confused at this hypocrisy of the grown-ups. Her thoughts are stifled as she doesn’t know if she should express herself or be quiet. Moreover, Nana and Maami constantly discuss the state of Adjoa’s legs to her consternation. Nana finds them too thin and long for a girl destined to become a mother. She is concerned about the fact that in future Adjoa’s thin legs may not be able to support strong hips for childbearing. Here, it is important to remember that Nana believes that the blame for being born with thin legs rests with Adjoa. It is squarely her fault for not being born with thicker legs and fleshy calves. The present skinny state of her legs is constantly lamented as they are considered useless. At such instances, Nana refers to her daughter’s poor choice of a husband who is not in the picture. It reduces the mother to helpless tears as she finds it unbearable to reflect upon her unfortunate marriage.

Adjoa is herself curious about the entire issue. Being a child, she doesn’t understand what childbearing legs look like and wishes to see them. Since she is perfectly capable of walking long distances and running fast, she doesn’t find any shortcoming in her own legs. She walks to her school daily. It is estimated to be five kilometres away from her own village but unlike others, Adjoa doesn’t mind walking so far because she enjoys her time at school. As an unlettered woman herself, her mother firmly believes that a school education would greatly benefit her daughter. She wants Adjoa to rise above ignorance and acquire knowledge for self- advancement. The major breakthrough in the story comes when Adjoa is selected as a runner for her age group, to represent the school in district sports events. The news is met with disbelief at her home and Nana marches to the school to confirm the veracity of it. To Adjoa’s great surprise, she finds that Nana takes up washing and ironing her school uniform daily indicating her acceptance of Adjoa’s new role as an athlete. When she wins the award for the best all- around junior athlete, Nana carries the trophy on her back with great pride. The undue concern regarding Adjoa’s thin legs is finally dispelled. Her thin legs may not support childbearing hips, but they can enable her to run very fast and be a good athlete. The trifecta of the three generations of women in the house ends the story on distinct notes of a newly found sense of pride and contentment. While Nana is proudly exhorting her granddaughter’s achievement, the mother is overwhelmed at her child’s success and absolution from the blame of birthing a girl with thin legs. Young Adjoa is perplexed at this change in the adults ‘behaviour but rejoices in her newly discovered agentive self.

Aidoo uses the seven years old Adjoa as the narrator of her story. Although she is a small child, she is extremely observant and intuitive. She watches the world around her keenly and presents a unique worldview to the reader using simple vocabulary. Her doubts and questions about her village life seldom elicit comprehensible responses from the adults. She is often given contradictory advice by Nana. She is alternately told to either be quiet or to repeat what she has already said for the purpose of adults’ entertainment. They find her ideas so preposterous that she is repeatedly told to ‘never, never, but NEVER to repeat that.’ The abrupt dismissal comes without any accompanying reason. She is never told why she mustn’t utter a few things and conversely why she must repeat others only to have adults laugh at them. Her predictable discontent at this duality leads to a vociferous internal monologue. Although Adjoa might have stifled her voice, her inner monologue is as perennial as ever. She tells herself, ‘when I think back on it now, those two, Nana and my mother, must have been discussing my legs from the day I was born. What I am sure of is that when I came out of the land of sweet, soft silence into the world of noise and comprehension, the first topic I met was my legs.’ The exasperation of Adjoa is legitimate because the discussion is so relentless, it might have started at her very birth. Her exaggeration does not make her an unreliable narrator. On the contrary, it is a mark of the oral tradition. She doesn’t see the world from the adults’ perspective and hence leads to a clearer account of the daily rhythms of the village life. Adjoa shows great insight in understanding that her small, all female family had divided opinion on the state of her legs. She finds, Maami and Nana arguing about her thin spindly legs.

It is common among the men in these tribes to have more than one wife at the same time. Polygamy is not just a prevalent custom; it is also a symbol of a man’s social status within the tribe. Spears, knives and clubs are their weapons and it is the duty of the men to provide protection to the families in the village and to also protect the boundaries of the village. Thieving is a common activity and since the cattle are a valuable resource they are at high risk of being stolen. With experience comes wisdom and the elders are highly respected for it. The old man had rightly warned the hunting party not to throw the spear which the thieves could aim back at them whereas Nyagar who was convinced within himself that the thief whose pockets he was going to search was dead could not think that the warm hand of the man could mean that the man was not dead but alive. Instead, he started imagining that there was something unnatural and ominous in the dead man’s hand being warm. He did not apply reason and common sense and was caught completely unawares when the dead thief fatally attacked him. Hunting in the primitive tribe is a community activity. Hunting is just hunting and it doesn’t matter whether an animal is being surrounded and hunted down or it is a man. In their primitive laws thieves and adulterers are regarded as animals and if one kills any of them he is not considered guilty of murder. The events which unfold their ignorance and hearts of darkness take place on a dark moonless night. The thief had failed to spot the bridge across the river in the dark night and fell into the hands of the blood thirsty crowd. In the story the white masters attempt to create a bridge between the laws of the ‘primitive society’ and the laws of the ‘civilized society’.

Drama, as we know, is quite different from a novel or a poem. We usually read a novel or a poem when we are alone. But drama is something more than simply words on the page –it is meant to be performed or enacted. The audience is an active participant in the theatrical presentation. So we respond to a play not alone but along with other members of the audience. It is quite another matter that other people will respond in their own specific ways. But then there are some plays which we do not see performed but read them as literature. How will we approach such plays? We must remember that playwrights usually expect their plays to be performed. It is for this reason that they present their themes through dialogue, action, music, song and dance so that the play is visually captivating. When reading a play we have to make full use of our imaginative powers. We must try to see the action and scenes as well as hear the voices of the characters. It is only then that we will be able to experience the play in its multiple dimensions: as ‘literature’ as well as ‘theatre’. You may face a slight problem trying to relate the English dialogues to traditional Marathi characters of the eighteenth century. As you know, the play was originally written in Marathi and comes to you in translation. You may have heard that the tradition of Indian drama is very old. It goes back to the Sanskrit drama of ancient India and encompasses contemporary Indian theatre in Hindi, English and the regional languages. Modern Indian drama is influenced not only by classical Sanskrit drama or local folk forms but also by Western theatre, following the establishment of British rule in India

A number of repulsive laws were passed to buttress the ideology and the policy of apartheid, engendering a state-sponsored trampling over of the black and nonwhite majority by the white European minority. The Population Registration Act (1950) prepared the bedrock by racially cataloguing people and made it possible for the white minority government to allocate discrete rights and privileges to individuals on the basis of their registration as white European, coloured South African, black South African or Asian. The Group Areas Act (1950) escalated the segregation by adding a spatial dimension to it. It clearly designated residential and professional spaces in urban areas for each racial group as well as restricted the entry and movement of members of other groups in areas not stipulated for them. Together with the Land Acts (1954 & 1955) and the Promotion of Bantu Self-Government Act (1959), the black South African majority was staring at an incredible proportion (more than 80 percent) of South African land being totally under the control and at the disposal of the white European minority, with no legal way for the blacks, the coloureds or the Asians to own these lands or possess properties on them. This jarring process of inferiorisation, delegitimisation, dispossession and dehumanisation of the black and non-white majority is both chronicled and satirised by a large number of South African authors, alongside the depiction of windows of resistance as glimmers of hope. “Let My People Go” (1962), the enlightening autobiography of Chief Albert Luthuli, delved into how theological apathy and political stranglehold were together squeezing the life-breath out of the blacks, reduced to slaves in the very land they belong to.

“July’s People” is set in a transitional period marked by political turbulence and clamour for a thorough social revamp. The intrinsically racist apartheid policy, though still functional, had lost all validity and was nearing its end along with the very regime, which had sanctimoniously brought into force such a disgraceful policy. The premise for the formation of a new government is explicit but its manner and aftermath are speculative. It is in this capricious context that Gordimer foresees an extremist insurgency by the blacks, pushed to the precipice, and how the resultant collapse of the apartheid regime metamorphoses the existing social equations between the non-whites and the whites. “The old is dying and the new cannot be born; in this interregnum there arises a great diversity of morbid symptoms”. But the interregnum here is not simply socio-political; it is also personal and ideological. Even for Gordimer as an author, the writing of July’s People is in that transitional phase of her life when she ceased to be the lighthouse of white liberal perspectives and consolidated her position as a staunch, unyielding proponent of racial justice as well as antiapartheid activism. In her essay ‘Living in the Interregnum’ she bluntly confesses: “I was a coward and no doubt shall often be one again, in my actions and statements as a citizen of the interregnum; it is a place of shifting ground, forecast for me in the burning slag heaps of coal mines we children used to ride across with furiously pumping bicycle pedals and flying hearts.. Her admission is not only indicative of her seriously heeding and engaging with locality, but it is also suggestive of how the mining town of Springs furnished the mental premise for realising the sort of change chronicled in the novel.

A man in short trousers arrives in the village with a battery-powered amplifier. The villagers drown in a night of unbridled merriment and get sloshed on the thin beer being passed around in plenty. The Smaleses refrain from the intoxicated celebration and return to their hut. Bamford and Maureen are horrified to discover that the gun and the boxes of cartridges are no longer there. Bamford feels clueless and powerless with no police to investigate the theft and recover the gun. This incident splinters his morale as he slumps into bed and “...at once suddenly rolled over onto his face, as the father had never done before his sons.” Divested of the bakkie and now the gun, Bamford’s crashing into bed is symbolic of the demolition of his masculinity, having lost both its external tokens. Maureen springs into action and looks for July near the bakkie. Her perennial reliance on him to retrieve things is reinforced once more as she demands that he has to get the gun back. July admits that Daniel has teamed up with the rebels and since he was the only one missing from the drunken revelry, it is rational to conclude that Daniel is culpable for the disappearance of the gun. Aggravating all of this, Daniel has now left the village and as Maureen keeps pressing July to do something, he loses calm attesting that he has had enough problems. She thunders back at him reminding him of petty household thefts from the past, which she found too distasteful to condemn, as her own rosy impression of his honesty would be torn asunder. At this moment, we witness the most emotionally fraught scene with July fulminating in his own language. Maureen cannot understand the language “...but she knows that what’s being said is an absolute rejection of her...not horrible, but painful.” Maureen is sapped of all figments of her liberal imagination and appreciation of July:
