# Supplementary material for: Dataset for classifying English words into difficulty levels by undergraduate and postgraduate students
Source: Data Brief. 2023 Oct 31;51:109744. doi: 10.1016/j.dib.2023.109744 (PMC10661753; doi:10.1016/j.dib.2023.109744)
Supplement: Supplementary file 6 [file mmc6.docx]

Later on Hester goes to the Governor's house along with Pearl. It is a very significant point in the narrative. Both the church and the state of New England are unanimous in persecuting Hester. There is a proposal to separate the mother from the child because a fallen woman is not supposed to be a good mother. Hester, however, opposes the proposal vehemently and urges Arthur Dimmesdale to speak on her behalf. Her words to the priest have a contemporary ring even for a reader of our time--"Speak for me! Thou knowest--for thou hast sympathies which these men lack--thou knowest what is in my heart, and what are mother's rights, and how much the stronger they are when that mother has but her and The Scarlet Letter". It is clear that Hester is asserting the mother's rights at the Governor's house where she has to confront singlehanded the hostile prejudices of male society in colonial New England. It has also to be noted that she is affirming the rights of a fallen woman to keep and bring up her child in a male-dominated society where both the church and the state marginalized such women and rendered them utterly powerless. You can easily imagine how forward-looking and almost prophetic the assertions of a fallen woman’s mother rights are in The Scarlet Letter!

Arthur Dimmesdale defends vigorously Hester's right to bring up her child and his vehemence creates a suspicion in Roger Chillingworth's mind. His reaction is brief, "You speak, my friend, with a strange earnestness" but it shows a strong doubt in his mind pertaining to the exact relationship between Hester and Arthur Dimmesdale.

A short story is defined as a self contained prose fiction or narrative about an incident or linked incidents evoking a particular effect and which can be read in a single sitting. It is shorter than a novel and revolves around a few characters. Bigger is not necessarily better. In the smaller space of a short story the central theme is often conveyed more poignantly and the characters are also stoutly built up. Small is beautiful. Writers can hone their skills of writing through writing short stories. However, short story as a genre received very little critical attention. This fact is true in the context of the postcolonial short story as well and in the postcolonial critical canon the short story has been systematically sidelined.

If we closely follow the characteristics of modernism it will be evident that there is a paradoxical relationship between modernism and tradition. On the one hand, modernism seeks to liberate itself from inherited tradition of values, ideals and cultural forms. There is a resistance to inherited culture, a complete break with the past. At the same time, as shown above there is a return to ancient myths from various cultures and religions with a deep sense of alienation, loss and despair.“ These are the two phases, positive and negative, of the modern as anti- traditional; freedom and deprivation, a living present and a dead past.” Modern imagination is both liberated and alienated. Thus modern writers in search of originality have gone back to ancient myths and traditions.

A language is the speech of a people. The history of a language is, therefore, the history of a people. English perhaps started as the speech of some Germanic tribes in Europe and through a series of changes brought about by migrations, invasions, conversions, settlement and colonisation assumed its present avtara. The Germanic speech group itself constitutes a branch of the Indo-European family of languages.

Hester retreats from the market to the lonely cottage outside Boston marks a new phase in her life. She is able to support herself and her daughter Pearl through her needle work. It is her aesthetic sense and her exquisite needlework that enables her to perform the duty of a single parent in a traditional, male-dominated society. The narrator has drawn our attention to "all the combative energy of her character” which enables her to serve the poor and the needy. There is a subtle transformation in Hester and the community that has persecuted her as the fallen woman begins to look upon her as a Sister of Mercy. The Scarlet Letter A that in the beginning appears to the community as a badge of sin and shame later on appears as a symbol of her calling as a Sister of Mercy.  In keeping with her status as a Sister of Mercy, Hester's dark, Oriental beauty also undergoes a change. The narrator's comment on this change is revealing indeed, "It was a sad transformation, too, that her rich and luxuriant hair had been cut off, or was so completely hidden by a cap, that not a shining lock of it ever once gushed into the sunshine"

Now the way the book winds up is this: Tom and me found the money that the robbers hid in the cave, and it made us rich. We got six thousand dollars apiece--all gold. It was an awful sight of money when it was piled up. Well, Judge Thatcher, he took it and put it out at interest, and it fetched us a dollar a day apiece, all the year round--more than a Body could tell what to do with. The Widow Douglas, she took me for her son, ad allowed she would sivilize me; but it was rough living in the house all the time, considering how dismal regular and decent the widow was in all her ways; and so when I couldn't stand it no longer, I lit out.

A leading black educator is reported to have said: "The white man may not intend nigger to be derogatory--but to the black man it is always derogatory and demeaning.' Ralph Ellison the author of The Invisible Man held that the white immigrants uncertain of their own identity seized upon "the presence of black Americans and used them as a marker, a symbol of limits, a metaphor for the 'outsider' Perhaps one of the first epithets that many European immigrants learned when they got off the boat was the term nigger--it made them feel instantly American."

It is absurd to assume, as has been the tendency, among a great many Western anthropologists and sociologists that all traces of Africa were erased from the Negro's mind because he learned English. The very nature of English the Negro spoke and still speaks drops the lie on that idea.

We need to be aware, at the very outset, that this is perforce a somewhat limited and hazardous task. It is limited in the sense that we will only be able to touch the surface of the vast ocean of their prolific writings, only have a peep or glimpse into their fascinating worlds. Each of these men wrote a huge quantity of prose: each of them, moreover, led exemplary and distinguished lives, which impacted considerably on the entire flow and direction of our cultural formation during a crucial time of our national awakening. That is, all three of these figures were active at approximately the same time, from the late 19th to the mid-20th century. Of the three, Swami Vivekananda was not only the eldest, but also the first to die. His brief life of less than forty years, however, left a blazing trail which the two other men, in their own ways, both followed and furthered. What is more, we'll also need to be aware that all three of these writers, though each is different and unique, shared a common outlook not just on India, but also on life. This outlook may be termed "national idealism," for the sake of convenience. What does "national idealism" mean? It means, simply speaking, two things. First, an attitude of reverence and respect for the nation that is India. This attitude was, by no means, uncommon to that period. As you know, India was emerging from centuries of subjugation in a new self-assertion. On the one hand, the struggle was to free India from British imperial rule. But, on the other hand, in order to accomplish this, Indians, with their huge diversities and differences, had to be welded into a modern nation. Cultural cohesion, therefore, was very important to the larger enterprise of nation building

Now if we pause for a minute and ask how come people in India decided that the realization of the Spirit or of the Self was the most important thing to do, that that is the purpose of our birth? What made the Indians think this way? It seems to me that long ago, the best minds in India discovered that it was not very difficult to sustain life in this subcontinent. It was not very difficult to take care of all of one's bodily needs and necessities. They lived on fruits and roots; they bathed in the flowing streams: they lived in harmony with nature. Similarly, it was not very difficult to make a hut a cottage, a hermitage. Nature provided enough. So they retired into the forest devoted themselves to contemplating the eternal truths. Most of the time and energy could be devoted to self-realization, to sadhana. This does not mean that our ancient rishis and munis were isolated from society. Instead, they interacted with the kings and commoners, guiding both, helping to uphold Dharma. They held the remote control of our civilization in their hands, letting the kings and courtiers to handle the mundane, material aspects of life. While other cultures thought that it was more important to build cities, to subdue enemies, to increase one's comfort and power, Indians recognized that all these activities were not as important as self realization. Without the latter, all human achievements were partial and transitory. I believe that these ideals are still present in our culture. Everyone laments that we are becoming more and more materialistic, yet as a civilization we have yet to admit that that is the supreme goal of human life.

The question of language is seen differently by the new writers. As far as they are concerned the language they write in is theirs, English is their first language. But this is English that has undergone many changes. But even this English cannot represent the complete daily life of any individual in India, where even the die-hard English speaker will have to use some other Indian language(s) in various social situations. If this is not taken into account, many nuances which can be exploited by the writer can be lost to her/him. Also, many Indians do not use English at all in their daily lives. How does the Indian English writer represent them? So even if English is the first language of the new writers they should be aware of the challenges that have always been there for Indian English writers. The Indian English writer is duty bound to create and use English that makes it impossible for the monolingual English reader to appropriate his/her experience with any degree of ease. Don't write like the English, and don't write what can be easily written in the Indian language of your region.

The early writers in English all came from areas which had the greatest British influence, namely the Bengal region, the western coast, and the south.

In most books of history written by Europeans, the continent of Africa is referred to as 'the dark continent'. This expression-the dark continent-was most probably first used by travelers and missionaries who happened to visit the African continent much before political claims on its territory were made and it was colonized by various European powers. The term 'dark' has been used for describing the continent of Africa because, firstly, these early travelers as well as missionaries considered as 'dark' anything mysterious that they did not understand much about. Secondly, and more importantly, these first visitors considered Africa to be inhabited by people who were primitive, uncivilized and savages. The colour of ignorance, as we know, is black. According to these early missionaries, the Africans believed in magic and other so-called irrational rituals and customs. Once again, magic is associated with black colour as, for example, the term 'black magic’ implies. These opinions, particularly about the Africans being primitive and uncivilized were accepted blindly by most Europeans who entered the continent either for trade or for colonization. In fact, the colonial administrations built upon this myth of primitivism of the Africans by stating that Africa had no history, no culture, and no past. The European powers used the excuse of 'civilizing' these savages for entering Africa and for staying on for the economic and political exploitation of its people. Thus was created the myth of the white man's burden which expression meant -on the face of it- that the white man had taken upon himself the onerous task-the burden-of "civilizing" the savage Africans.

Land grab was not the only problem brought about by the colonial policy of European settlement. The settlers wanted a constant supply of cheap or free labour to work on these farms and with the African reluctance to work for outsiders in these, European farmers, found it increasingly difficult to obtain cheap labour. The African's reluctantly was due to the fact that their basic needs were provided by the subsistence economy and moreover, they did not want to work for the colonialists. In those cases where African settlements were part of European acquired lands, the Africans were declared squatters with permission to cultivate a small plot of land on the farm premises- -Shamba-and to keep members of the family as well as a few cattle. Appalling working conditions and severe restrictions on both tile physical movements of the squatters and also on the number of cattle they could keep and the kind of crops they could cultivate were definitel3- oppressive. Moreover, the wages were abysmally low. They were subjected to most cruel punishments on the flimsiest of excuses. In fact, their plight in many ways was worse than that of their brethren who had been sold as slaves in the Americas by the Arabs and the Europeans. The result was that as in other countries of Tropical Africa, labour force in Kenya was created by 'methods of extra economic coercion,'

In fact, the settlers, with the help of successive government legislations seized more than 7.6 million acres of most fertile land. Yet even close to the end of the colonial period only 18% of this land was cultivated while millions of Africans strived to eke out a living in highly congested reserves.

A writer sees before him the immediate, concrete reality but he does not, unlike the historian, remain on that plane for long. He moves away, going beyond the objective concreteness to what may be termed as the level of artistic concreteness. At this level of artistic concreteness, the miter creates a world of his own, a world in which

through the creation of individual characters--imaginary or real, or both—he investigates the social being of man and the role of certain sections of society in history, the conflicts of their interests and the nature of their clashes and cleavages. It is through the study of these characters that a writer gives us an idea of the mentality of a people, their moral attitudes, their ideals and aspirations. The writer distills the historically significant phenomena and also reveals the links between literature and society. Each of these characters gives us a glimpse into the various stages of socio-political development and evolution of epochs. It is, therefore, by going 'beyond' that a writer rises to a universal level where a new artistic reality is born, a reality which is not very different from the objective one which delimits the range of history but which is aesthetically interpreted. Depicting reality, therefore, is a process of creation and not of imitation. It is an act which portrays life in motion and in a perspective that through the depiction of the particular, expresses simultaneously the regular, the universal and the typical. To obtain this level of realistic depiction is, however, by no means an easy task and a writer, in its pursuit, passes through various stages: at the first stage, a writer singles out and records what he believes tc be most important and characteristic out of the diversity of facts and the chaos of everyday life.
