# Supplementary material for: Dataset for classifying English words into difficulty levels by undergraduate and postgraduate students
Source: Data Brief. 2023 Oct 31;51:109744. doi: 10.1016/j.dib.2023.109744 (PMC10661753; doi:10.1016/j.dib.2023.109744)
Supplement: Supplementary file 7 [file mmc7.docx]

There are critics who think that the greatness of an artistic creation lies in its richness, ambiguity, and interpretability, and that it is both futile and somehow wrong " to search for the correct interpretation, the one which the author intended' wrote E.H. Gombrich in his essay 'The Evidence of Images' and went on to affirm 'I do not hold this view. I do not believe that any interpretation is sure and infallible, any more than any other hypothesis can be. But I do think that we can try as historians to restore the original context in which these words were intended to function and it is always worth-while to venture upon this perilous path.. . ' In his essay Gombrich picks up problems of interpretation of visual images for military intelligence, ornithologists, communication scientists and art critics and shows the centrality of interpretation to the business of living. However, you may think that interpretation is a western fad and a byproduct of scientism. Let's allude to two episodes in Sanskrit literature. This first one is from Kalidasa's Sakuntala. King Dusyanta, as you know, lost his lady love due to a curse. While the Xing was once looking at a painting with three women Vidusaka comes in and the king asks him who according to him is Sakuntala. Vidusaka replies, 'she who is leaning rather wearily against the mango tree, its leaves glistening with the water she has thrown over it. She extends her arm with infinite grace, her face is slightly flushed with the heat and flowers entwine her streaming hair to fall together over her shoulders. She must be Sakuntala and the others her friends or maidservants'. (We can compare the portrait to a lyric which celebrates a single intense moment, the painter in Sakuntala took a single moment in his/her subject's life and immorlalized it with his brush just as a poet does with his pen.)

In one of his last poems written in 1938 called 'The Statues' the Irish poet W.B. Yeats. (1 865- 1939) marveled at 'The lineaments of a plummet measured face'. As you how masons work with plummets which is a plumb or ball of lead attached to a string for testing perpendicularity of wall, etc. And yet the 'plummet measured face' has its distinctive’ features or lineaments. Salamis, which you may locate on a map of Greece, was the site of the rout in 480 B.C. According to Herodotus (5th B.C.) the Greek historian who had participated in the war and left an account of it, the armies of the Persians were fantastic; their might unchallenged. However they were defeated by the cooperation of Athens and Sparta. Salamis is seen here as a symbol of the victory of mathematics, calculation, numbers over 'vague immensities' and the proverbial Asiatic grandeur. We are reminded of the sea battle at Salamis by the 'many headed foam' in the sixth line of the quotation above. In the same line Yeats cunningly slips in the name of Phidias, who was perhaps the greatest artist of ancient Europe. His colossal statue of Zeus at Olympia in the south-east of Acropolis wrought in ivory and gold over a core of wood was the most famous statue of antiquity. He had also contributed three statues of Athene on Acropolis. One of them was wrought in ivory and gold. He had also probably designed and certainly supervised the construction of the frieze of Parthenon We may, may not or only partially agree with Yeats's observations above on 'Asiatic vague immensities ' but we cannot deny that pieces of art, or any work in politics or warfare for that matter, are human contrivances of planning with the help of cold concrete facts -be they words, or colours or rocks and mortar or people and locations.

**Prosody**: That part of grammar which deals with laws governing the structure of verse is called prosody. It encompasses the study of all the elements of language that contribute towards acoustic or rhythmic effects, chiefly in poetry but also in prose. Ezra Pound called Prosody "the articulation of the total sound of a poem". However, we how that alliteration (the rhythmic repetition of consonants) and assonance (repetition of vowel sounds) occur as much in prose as in poetry. Besides assonance and alliteration rhythmic effects are produced in poetry as well as in prose by the repetition of syntactical and grammatical patterns. However, compared with even the simplest verse, the "prosodic" structure of prose would appear haphazard and unconsidered.

**Metre and Metrics**: Metre measures the rhythm of a line of verse. The word metre derives from the Greek word *metron* which means 'measure'. Traditionally metre refers to the regular, recurrence of feet. According to the Hungarian-American linguist John Lotz (b. 1913), 'In some languages there are texts in which the phonetic material within certain syntactic frames, such as sentence, phrase, word, is numerically regulated. Such a text is called verse, and its distinctive characteristics meter. Metrics is the study of meter. A nonmetric text is called prose.' In the words of Seymour Chatman (b.1928) 'Meter might be defined as a systematic convention whereby certain aspects of phonology are organised for aesthetic purposes. In order to find out where the accent falls we scan a line.' 'Like any convention' Chatman goes on, 'it is susceptible of individual variation which could be called stylistic, taking "style" in the common meaning of "idiosyncratic way of doing something”

Quantity in the present context refers to the time we take to pronounce a syllable. It is a product of the duration for which we pronounce the vowel at the nucleus of the syllable. For instance you can pronounce "sweet rose" in various ways shortening and lengthening the vowel sound as you please. This variability, however, would hinder communication between the poet and you as the reader. Now if you compare Sanskrit or Hindi for that matter, with English you find that you cannot exercise your discretion in lengthening or shortening the vowel sound or the quantity of the syllable in the two Indian languages. They are predetermined by the linguistic system of Sanskrit and Hindi.

The quantitative metres dominated Greek and Latin poetry because they are highly inflected. (To inflect a word is to change its form at the end according to its peculiar, case, mood, tense and number. For instance we can say that "child" and "boy" inflect differently in the plural.) The inflection promoted the construction of long, slow paced lines because those languages supported the alternation of the long vowels in the roots and the short ones in the inflections. English which lost most of its inflections in the 15th century, unlike German, is less hospitable to the quantitative metres.

**Scansion**: In general parlance, to scan is to look intently at all parts successively. Radars cause particular regions to be traversed by a controlled beam. In prosody scansion refers to metrical scanning of verse. When a unit of verse - a foot, a line or a stanza - is scanned with the help of symbol's the metre can be seen as well as heard.

Rhythm is to borrow Plato's words, 'an order of movement' in time. We generally speak of rhythm in connection with poetry or music. However, you must have heard people talking of the rhythms of nature or even biological rhythm. -Perhaps periodic repetition of a certain pattern is the sine qua non of rhythm. All the arts-painting, sculpture, and architecture - have their rhythm. Here, however, we will talk of rhythm in the context of poetry only. Above you studied about a variety of acoustic effects in poetry such as metre, rhyme, alliteration, onomatopoeia, etc. They contribute to the rhythm of a poem. Prosody which states into account the historical period to which a poem belongs, the poetic genre and the specificities of a poet's style goes closer to the rhythmic aspect of a poem. For instance, quantity (or vowel length) is a rhythmic but not a metrical feature of English poetry. This is because English does not impose any strict regularity in quantity as it does with respect to stress. For example in 'sweet rose' the vowel sounds can be lengthened or shortened at will. This cannot be done in many Indian languages. However, the lengthening and shortening of the vowel sound does affect the rhythm of the poem. Similarly, the rise and fall in the human voice especially in reading poetry which is called cadence is a rhythmic not a metrical feature. Many other factors contribute to the rhythm of a piece of verse or prose. Grammatical features are some of these. According to Yeats, "the arts have already become full of pattern and rhythm. Subject pictures no longer interest us." In this context he refers precisely to Degas, in Yeats' opinion an artist whose excessive and obstinate desire to 'picture' life - "and life at its most vivid and vigorous" - had harmed his work.

Criticism has often been described as the soul's adventure among masterpieces - and this course which for you is an adventure of critical appreciation began with an appreciation of two portraits that also symbolically meant to tell you about this course. Besides, each block will have one or two copies of paintings that are meant to serve as frontispieces and also visually tell you about the age. Just a few comments are offered on them in the introduction to the blocks. You may explore further on your own because it has been recognized since time immemorial that proficiency in several arts is necessary for specialization in any one. Did you read the epigraph of this course? It: can as well be a desideratum for you. In this unit we examined in the first place the thing called literature, especially poetry in somewhat abstract terms. In the second place we examined the prosodic aspect of poetry. Finally we showed how the various aspects can be put together in our critical appreciation of a poem. In the last major section we have done for poetry what in the previous unit we did for portraits - we critically appreciated a poem. This is what we expect you to be able to do on this course. Critics say that the evolution of the rhythm of a language tells us about the cultural evolution of the people, their changing and evolving consciousness. If this is a tall claim I leave you to decide for yourself. At the M.A. level we I did not consider it necessary to describe the genres such as lyric, epic, ode, etc. or figures of speech such as simile, metaphor, synecdoche, metonymy etc. You should consult a dictionary of literature in order to discover the terms of art as and when you feel the need to do so.

The Elizabethans have immensely enriched the Greek tragic form both in their adherence to dramatic form and in the liberties they have taken from the rigorous discipline of the Greek dramatic art. The Elizabethan tragic protagonist is an Aristotelian hero, usually of a noble birth, blessed with outstanding qualities but suffers from a serious tragic flaw or hamartia in his character that sets the play in motion. The Chorus plays the introductory and summative function as in Greek Drama. Plot is a major element as in a Greek Drama using the devices of peripeteia and anagnorisis. The interest of the audience is sustained by the spectacular action and dramatic irony whereby the audience knows the predicament of the protagonist that the latter fails to understand. The plot leads the protagonist to a tragic recognition of his weakness while the audience gains a cathartic experience of the feelings evoked in the course of the play. The Elizabethan drama abounds in the number of dramatic characters in a play while their number was limited in the Greek Drama. The dramatic unities are followed more in their breach by the Elizabethans who try to encompass a larger and larger framework of time and place, for their renaissance aspirations drive them to boundless action. Similarly, a zest for a diversity of experiences always haunted the Elizabethans making it impossible for them to stick to the Greek dramatic distinctions of tragedy and comedy. For the Elizabethans, tragedy is not a restrictive view of human excellence or weakness as the Greeks are often inclined to present but an affirmative view of human aspirations whose pursuit brings a glory to the definition of man. Struggle, conflict, suffering and failure may be the inescapable attendants but the human spirit is not stifled in its stifled by what attends to them.

A work of art is an organic whole. It is one work. It has one voice and that voice must speak for the whole work. It must speak collectively for every part that makes up the whole that the work of art before us is. It is not the same thing as suggesting that a work of art must or can have only one meaning. Great books have a tendency to speak to each reader in a different mood and meaning and impart a different significance. In fact, each reader finds himself responding to a different significance each time he reads a great work of art. But that one meaning must answer every question that that text must raise, and justify all that happens, for instance, in a play, in its every word, gesture and action. In other words, all interpretations proffered as meaning of a play must derive validity from the text of the play itself. What we are not looking for is the most authentic meaning -how are we to arrive at its authenticity. One way of looking at this issue is to remember that once a work of art is written the author is merely a reader of this work; one more reader of this work. Maybe a principal, even the principal reader of this work, but merely a reader nonetheless. Once a text sees the light of the day-or the darkness of the print, if you like-it becomes an angle in the triangle with the author and a common reader or a professional scholar as the other two angles. The interaction between these three angles offers endless possibilities of intellectual pleasure and profit. But our ultimate focus is the work of art that is before us. The meaning that we are looking for is the one that satisfies a reader the most and explains in every way the complex entity that the work of art in question is.

When we, along with Hamlet, finally arrive at the end of the play and gain our share of the wisdom, "The rest is silence!"-we suddenly realise that in one sense Hamlet progresses through a whole series of events, actions and ruminations to grapple with the significance of the absence of silence. Language seeks to make possible apparently that is not possible through silence-communication. And in Hamlet characters constantly question the wisdom of relying upon words. Words fascinate them, and there is an ongoing debate in the play about the use, abuse and futility of resorting or not resorting to the medium of words. One of the major issues in Hamlet appears to be: Does language stand in polar opposition to action? Is it irreconcilable to action? Can it, or can it not, further or motivate action? The philosophical relationship between thoughts, words and deeds, thus, turns out to be a major issue in the play. Words stand out in our recollection of Hamlet as much as vivid visual images. One of the intriguing things about Hamlet is the fact that everybody remembers words from Hamlet -more than any other play by Shakespeare or anyone else. Everyone can recollect, quote, or recognise quotations from Hamlet. If almost all these words that linger in our mind long after we finish reading the play belong to Hamlet, it is also because the prince who speaks these words is much better with words than with actions. To justify his procrastinating taking action he plays with words, argues with them, through them, for and against them--of course in words. He repeats. Repetition of words and phrases occurs so frequently in the speeches of Hamlet as also in those of other characters that one suspects that an ongoing march of words is used to reflect one of the major themes of the play, procrastination.

Language plays a major role in the definition of a character's trait in conditioning our response to him. We must appreciate that great popularity that Shakespeare and his characters have enjoyed over the last four centuries owes itself in a major way to the language of his plays. What the characters say is important, because they linger in our minds for what they say as much as for what they do or feel or suffer. But the way they say what they say is of paramount importance. The manner and method of a speaker affects the response of the audience to what they hear. Theatre-goers respond not merely to the meaning of words, but also how the words are conveyed to them. Shakespeare's choice of metre, rhythm, imagery and, of course, diction, determines how we respond to what the characters say, and to the characters themselves. Ultimately this affects-enriches--the total experience of interacting with a play on stage or on page. The meaning of the word is important but attention should be paid to what goes into making the meaning of words effective communication and manipulation of audience response. , An interesting aspect of Shakespeare's use of language is the fact that certain linguistic features are meant to be appreciated as rhetorical devices for their own sake and not merely as starting points for generalisations for the larger context of the text. Hamlet is rich with imagery. Vivid descriptions, carefully chosen words and phrases and used with deliberate effort and intention provoke us to see imaginative reconstruction of what is otherwise mere communication through words on a page. They add to the pleasure of interacting with a text. A quibble is to Shakespeare what luminous vapours are to the traveller; he follows it at all adventures; it is sure to lead him out of his way and sure to engulf him in the mire.
