# Supplementary material for: Dataset for classifying English words into difficulty levels by undergraduate and postgraduate students
Source: Data Brief. 2023 Oct 31;51:109744. doi: 10.1016/j.dib.2023.109744 (PMC10661753; doi:10.1016/j.dib.2023.109744)
Supplement: Supplementary file 8 [file mmc8.docx]

While reading a novel, we may feel that we have been transported to a different world with its own laws, rules and regulations. Towns and villages, markets, streets and pathways hold out as actual places' with their distinct colouring and feel. Yes, the emphasis is on actuality. Not only are the people shown as speaking with their very own mannerisms, but ordinary information about their appearance, condition, opinions and states of mind also is imparted. This second aspect of the writer's practice implies that the describing person, the novelist, has an opinion and a point of view according to which he judges without much scruple the actions of the different characters selected, consciously and with an ostensible purpose, for presentation. The judgment of the writer is biased as all judgments are. The biases obviously indicate that the writer is totally immersed in the overall fate of the characters as well as the effect of their behaviour on the life and nature of the society. The writer can be seen as a responsible member of the actual society of that time as well as the society reflected in the novel. The remarks of the author meant clearly for sharing with the reader, lend authenticity to the description in the novel and make the reader accept it as a truthful account. This leads to a state in which the reader is strongly drawn into the ethos of the world of the novel. The reader may feel that he is witness to an actual happening in which real people have been involved. The words in the text do not merely signify something outside of them. Instead, the words are there on the page as a picture or pictures which introduce the reader to their world and bind him to its specific aspects. There is no wonder that the reader of the novel would get fully absorbed in the goings on of the world chosen for representation in the work. This is what I mean by the novel as a realistic form.

Fiction or fictional has come to acquire such strong affinities with the novel that we use e two synonymously. Walter Allen in his book The English Novel has drawn our attention in this regard to the issue of artistic representation—the way a writer gives ape to an experience in her/his work. Characters in a novel symbolise specific attitudes a given society and the writer conveys through them those significant impressions which she or he has gathered from the surroundings. Characters and social impressions merge into each other and the end-product strongly binds us to the represented action. But the writer does not merely 'gather' impressions from life. What happens is that impressions precede characters and are in fact moulded and re-made into characters by e author. In this sense, they are truly fictional — moulding and remaking imply that the author's imagination has been at work in an intense manner. There is also the problem of plausible, life-like situation that the writer is supposed to invent. This means that characters in the novel cannot be constructed and rendered flesh and blood unless they placed in identifiable circumstances of our own world. The men and women in a work of fiction become our links with the period in which the writer has lived and stand r those actual trends that existed at the time. Through Allworthy, Western, Jones and Blifil in Tom Jones, for instance, we gain close familiarity with the developments in eighteenth century England. The process is complex but the truth is quite simple. In a peculiar way, the actual circumstance, the society of a period becomes a necessary component of fiction. Fiction becomes significant history. That is how the line between e imaginative and the real gets blurred and history intrudes inevitably into fiction.

The question posed here is whether fiction comes to gradually resemble history, or to put another way, history becomes the all-important subject of fiction. We can take the argument onto another plane and say that around the eighteenth century in England, history becomes a matter of vital interest for the common writer who sets out to do justice to it by focusing upon the behaviour and problems of ordinary men and women. However, the novel is different from history in one important respect. History as we see is a long continuous process without a clear tangible beginning as well as an end — it goes on unfolding itself beyond its specific actors of a period, its men and women who a active within it to influence and change it. On the other hand, the novel begins at a particular point of life in society as well as ends at another point. Those two points in the novel, recognised and chosen by the author are extremely significant, because between them lies that segment of social life -captured as it has been through words -which vibrates with meaning at every turn and also contains within itself a totality and a certain truth. It is a significant difference between history, the life of actual people at a given time, and a literary work. In the examination, students are generally asked to comment on the ending of a novel and tell the truth that has been constructed with its help. Why? Because it is assumed, and rightly perhaps, that the end matters in terms of the lesson which the novelist set out to convey to the reader. Replace the word "lesson" with the word "moral" and what we have is a fable which has to establish a useful aspect of human wisdom relevant to the, period in which the writer lived.

The reader gains this wisdom by virtue of arduously following the course of events depicted in the novel and sees that the author consciously took him on a specific journey in imagination. The same thing can be perceived in an account of history but with less emphasis since the historian is much more answerable to the actuality of events, the socio-historical reality of the period under study. In history, moral lessons can be noticed as merely scattered, and the person, the historian, if he chose to clearly underline these morals, can be accused of violating laws of objectivity. He may face the accusation of allowing subjective biases to play the decisive role in the presentation of the historical account. Yes, there are lessons in history, but they are the tentative creations (of course, no useful study of history is possible without them) of the perceiver 6r the interpreter, not of history as such. For instance, a specific 'understanding' of history can be countered by 'another' understanding. You can see contrasting lessons conveyed by another interpreter of the same period in history. This is because history is no single person's or group's creation - in fact, being a bigger continuum; it is not the creation of any person, group or, even, the whole society of the specific period. Simply taken, it is found there when we are born and it would, hopefully, be there when we die. In contrast, the novel is an author's creation - it entirely belongs to him or her. If the individual so wished, the writing of the novel could be indefinitely deferred or the idea altogether discarded. Such is the grip and bind of the author on the novel, on its writing. Starting from the idea of the fictional piece, the author gives it slant and direction chooses from. This means that the shaping of the novel involves a great deal of flexibility.

There are a large number of books on the subject that provide good information about prose works in the sixteenth and seventeenth centuries. The idea in these books is that the prose works of the earlier period can be clearly linked up with the novel in the eighteenth century. The common point between the two seems to be prose. Then, there are the stories of discovery, exploration and adventure, which also have laid claims to parenting this modern literacy form. It is suggested that the spirit of curiosity necessitated a loose fictional form which provided enough scope to the writer to collect information as well as to question, analyse and assess the new material. These stories centered around 'the wandering rogue,' a rootless, un-tied persona whose fascination for new and unknown places could hold immense appeal for the reader. Add to them the imaginary, totally 'fictional' pieces written by their authors in different countries of Europe to entertain the reader, taking him or her on an imaginary voyage to the world of mystery, wonder and magic. In these, nothing 'real' was intended for projection, their fundamental motive being to give pleasure. Curiosity, suspense and story-telling were supposed to bring these writings closer to the novel. In this context, all one can say is that important as these imaginative efforts are in their respective languages and periods, they scarcely enlighten us about the emergence of the novel? All that has come in the wake of such a venture is mere guesswork. In fact, the fault in such a genesis-tracing exercise is that it is based on the erroneous concept of literary history. The term 'literary history' denotes that there is a direct linkage within literature between works written in the past with those written later, arid that, in a manner of saying, literature produces out of itself.

Comedy in the eighteenth century differed immensely from that in the seventeenth century. It became lighter in vein and dealt with those issues which could be easily resolved. Take the case of social manners under whose overall perspective questions such as remarriage and love were considered by the writers. The man and the woman together took the decision to marry and thus set at naught the pressures of family and society. As a consequence of this emphasis in the eighteenth century on decision-making by the individual, norms and principles of orthodoxy came under severe criticism. One of the reasons why an ordinary person because associated with heroic qualities such as courage and fearlessness was that an important segment of society, the middle class to be precise, stood to gain from protest and rebellion since that weakened the hold of the privileged sections on social behaviour. Under this logic, marriage became a means for the middle class to question the values and norms espoused by entrenched interests. The focus on social manners takes us away from the serious questions of work, shelter and upkeep to be provided by a society to its members. Only those who have solved the problems of bread and butter think of evolving a code of behaviour. The issues of virtue, goodness, morality and kindness which fall under the category of ethics and manners are of great interest to the progressive upcoming sections. Further, the discussion of manners suggests that the members of this group have become individually capable of improving their behaviour that they have merely to take a close look at their norms and principles in order to adopt a strategy to execute progress and improvement. In this sense, the improvement in manners is primarily a question of active choice.

But there is another format that of the comprehension of a literary pieces by the "common reader," the educated sections in the larger society, as different from the "specialist reader," the student of literature and the critic. The common reader may not be "professionally" interested in seeing those nuances which presumably take our response to a higher plane; he or she may instead seek a direct link of the work with the world surrounding him or her. This happens many a time in the case of the works which reflect specifically on contemporary issues. The common reader may feel that the book in question - the poem, the novel, the play - contains a message and a statement about the actual situation of the time. The question is: How do we as serious "uncommon" readers of literature deal with such an attitude? In my view, the idea of literature as carrying a message should always be cherished, though we notice that most of the criticism coming from the western academe has discarded it. The academy in the first world, that is how they have to be called by us, tends to divide the human creative-intellectual endeavour in separate compartments economy, politics, philosophy, print and audio-visual media, ideology, literature, theatre, linguistics, the arts, etc. Under this scheme, the message has been assigned to politics, ideology or the media. Literary criticism, on the other hand, has come to gradually constitute the "internal areas," the linguistic-textual aspects of a book. But can we, the members of a third world society which stands deprived of even the basic means of subsistence, afford the luxury of a class-room or seminar format? In fact, Raymond Williams reminds the reader in the developed world of the west that literature should be firmly placed "in society."

To mind, characters afford important clues to the author's attitude and response. They enlighten us about the way the author's mind works in the process of understanding hum personality within a particular system. But before I comment on a character or a set of characters in Tom Jones from the angle of human personality, I wish to state that a character in a work of fiction is always a symbol, a concretised pattern of social behaviour. What I imply is that social behaviour can be a good subject of comment – you can call a particular behaviour good, bad or just acceptable. This goes against the notion of that character who cannot be analysed except in psychological terms An individuality captured in a novel has nothing to do with a flesh and blood human being – it is a represented individuality, not an actual one. Let me explain further. A character's representation or what is called characterisation reflects essentially upon (tells about) its representer or creator — the author — and not upon that 'personality' which a novel appears to contain within itself. Arnold Kettle struggles with this idea throughout his two-volume study An Introduction to the English Novel. But Kettle does not resolve the dilemma between what he calls "life" and "pattern" and merely settles down to accept what he considers two separate types of fictional works under the categories of "life" and "pattern." Kettle has argued that there can be a certain type of character which engages the attention of the reader in realistic-experiential terms – the reader feels the way the character does – and that the reader enters the emotional world of the character. For Kettle, this is "life" as captured by a novelist. Kettle's category of "pattern" the opposite of "life” stands for the author's moral or social viewpoint.

The "individual" in a character could means two things: firstly, it denotes that sensuousness which draws the reader close to the character, establishes an imaginative link between the two and the reader "becomes one" with it. Authors achieve it through the psychological projection of characters in which not just the decisions taken but the processes through which the particular person reached his or her resolutions are communicated to the reader. There is a whole aesthetic argument based upon a character's "individuality" and "sensuousness" which certifies that character's, or even the whole novel's, authenticity. This, as I see it, is a narrow view of the concept of "individuality." If we adopted this view, we would be constrained to believe that Fielding's novels, and the characters in them, lack that verve as well as delicate sense of feeling in a situation which is the hallmark of intensely human writing. More, we might even visualise opposite polarities between the typical and the individual, the former standing for the social and the latter for the human. The second meaning of the "individual" has greater validity, in that it points towards the particular phenomenon of which a character is a part. In fact, we cannot separate a character from the larger phenomenon under whose specific rules it operates. Fiction criticism should go into this relationship between the characters and the phenomenon and find out the way in which the two "create" specificity together. Thus we see that the peculiar pattern of events presented by the author may or may not impart "sensuousness" and "authenticity" in human terms to its actors, it may not show them as "breathing" and "living," and yet engage the reader's attention in its descriptions because of the close identification the presented pattern may establish between the reality of the work and that of the reader.

Heathcliffs socially unacceptable behavior can be explained from a number of perspectives. Physiologically, we may see him as a traumatized child who grows up feeling neglected and bullied. A deeply hurt psyche, which on adulthood seeks not only revenge but is obsessed in an infantile way with the object of his love. Frustrated in love, he unleashes hatred. Heathcliff has a fractured psyche, and the strange circumstances of his childhood, makes his adult personality deformed and perverse. He is also the third angle to the love triangle of Catherine, Edgar and himself. He is deprived of his love, not because he is rejected by Catherine, but because he lacks social status and sanction and this is something he rebels against with vengeance. Heathcliffs behaviour can be explained sociologically if we consider the kind of injustices that are, perpetuated on him. By birth he is a social outcast for no fault of his. Catherine is accepted at the Grange but Heathcliff is not. In the Earnshaw household he is repeatedly demeaned and wronged. It can be argued that such victimization would beget violence of the kind that Heathcliff uses as an adult. Social oppression was tolerated by the 'sullen patient child' who was 'hardened perhaps due to ill treatment' and could stand Hindley's blows 'without winking or shedding a tear'. When Heathcliff wreaks his revenge, it is the pent-up anger of past injustices that makes him do what he does. The society, does not accept such individualized retribution and our narrator, Nelly does not approve of it. The artificiality of the civilized world as represented in the well-nurtured potted plants and flowers at the Grange is contrasted with the wild naturalness of the moors where Heathcliff and Catherine can abandon themselves, liberated from the oppressiveness of the same artificial and structured civilized society.
