# Supplementary material for: Dataset for classifying English words into difficulty levels by undergraduate and postgraduate students
Source: Data Brief. 2023 Oct 31;51:109744. doi: 10.1016/j.dib.2023.109744 (PMC10661753; doi:10.1016/j.dib.2023.109744)
Supplement: Supplementary file 9 [file mmc9.docx]

An interesting thing to notice is that if a human child is brought up m isolation, he does not acquire language, whereas birds reared in isolation sing songs that are reconisable. Human beings require a long exposure to language in order to acquire it. This does not mean that human language is totally conditioned by the environment. According to Chomsky, human beings are born with an innate 'language acquisition device' (LAD), but environment plays an important role m triggering this innate ability. We should note that every normal child learns an extremely complex grammatical system before she is 3 years old. Language IS certainly one of the greatest wonders of human societies; it cannot be accomplished unless we were endowed with an innate language faculty. So we can say that although both humans and other animals seem to be genetically predisposed to acquire language, it seems in humans, this latent potentiality can only be activated by long exposure to language, which requires careful learning. Most animals can communicate about things in the immediate environment only. A bird utters its cry of danger when danger is present. It cannot give information about a danger which is removed in time and place. Human Beings, on the other hand, can communicate about things that are absent as easily as about things that are present. This phenomenon is known as displacement. It occasionally occurs in the animal world, for example, in the communication of honey bees. If a worker bee finds a new source of nectar, it returns to the hive and performs a complex dance in order to inform the other bees of the exact location of the nectar, which may be several miles away Human beings can cope with any subject whatever, and it does not matter how far away the topic of conversation is in time and space.

Most animals have a fixed number of messages which are sent in clearly definable circumstances. For example, a North American cicada can give only four messages and a male grasshopper has a choice of six. Research conducted on dolphins, birds and bees has also shown that they are unable to say anything new. Human beings, on the other hand, can talk about anything they like. They can produce and understand utterances which they have never produced or heard before. It is also not necessary that the same situation would make them utter the same thing each time. So we can say that creativity is an important characteristic which distinguishes human communication from animal communication. Animals that use sound signals for communicating have a finite set of basic sounds. The number of basic sounds varies from species to species. Cows, for example, have less than ten, whereas foxes have over thirty. Most animals use each basic sound only once, or occasionally few simple combinations of these basic sounds. This means that the number of messages that an animal can convey is almost limited to the number of basic sounds that that animal possesses. In contrast, human language works very differently. Every language has a set of thirty to forty basic sounds which are called phonemes. These phonemes are generally meaningless in isolation. Imagine a person uttering the basic sounds 'a .. k..u..t..v..r..l..j..h... Do you think it would be possible for this person to convey any meaning? These basic sounds or phonemes become meaningful only when they combine with each other in accordance with the rules of a 1anguage.So we can say that human language is organised into two levels or layers, i.e, a layer of individual sounds which combine with each other to form the second layer of bigger units like words. This kind of organisation into two layers is called duality or double articulation.

As mentioned earlier, structural linguistics owes its foundational debt to the great Swiss linguist Ferdinand de Saussure. His insightful observations on language as a system and his treatment of language primarily as a social phenomenon became the guiding principle for structural linguistics. The central principle of the Course is that a well- defined subpart of language called Langue can be abstracted from the totality of speech. It represents the abstract system of structural relationships Inherent in language - relationships that are held in common by all members of a speech community. Since langue, according to Saussure, forms a coherent structural system, any such approach to language which is devoted to explicating the internal workings of this abstract system is referred to as structural linguistics. The structural approach to the analysis of language is not only concerned with explicating the internal workings of Langue, but it also involves the segmentation of utterances into elements in terms of two basic and complementary relations: syntagmatic and paradigmatic. The former looks into those elements which combine to form a larger unit, while the latter tales those elements which call be substituted for another in a given context. In a structuralist description of language both an inventory of the linguistic elements of the language under analysis and statement of the positions in which the elements occur are taken into consideration -- the former refers to discovery procedure and the latter refers to distribution. The 1950s in the United States witnessed a spate of activities in structural linguistics with a distinct Saussurean heritage. Later, structural linguistics in America took on its distinctive cast and entered the period of its great success. Structuralism in the United States grew independently to that of its European counterpart. Their interests were different and they even differed in their understanding of the term 'structure'.

We often come across contradictory viewpoints about the relationship between language and thought. The issue has been examined for centuries form different perspectives including those of philosophy, biology, linguistics, psychology and logic. Some people believe that language is primary and makes thought possible. It is absolutely central to our thought processes; it neither only helps us to understand the thoughts of other people and express our own but also structures our thoughts in a variety of very complex ways. We see what our language tells us to see and we express our thoughts in ways that are allowed by our language, Language in this sense is constitutive of thought and defines the limits of our thought. This we may regard as the cognitive view of language. According to several other people though comes first, Language is only a tool for expression; a means of communication, in this communicative view of language, language is seen merely as a conduit through ' which thoughts are articulated. Thoughts and ideas exist independent of language. It is also claimed that a variety of thought is possible without language. Several important human activities including sculpture, dance, music, painting, etc, involve some of our highest thoughts but may be completely independent of our language. It is possible to argue that dance and music have their own language but it may be significantly different from natural human language which is rooted in a pairing of a lexicon with a set of syntactic rules. Again, many people will argue that dogs and call have emotions, understanding and thought. They may also have some kind of language but as compared to human language it is extremely limited in a variety of ways. It is possible that there is an element of truth in both these positions because nobody will deny an intimate relationship between language and thought.

Many people have found the Sapir-Whorfian hypothesis very attractive; they feel that language determines their thought patterns in important ways. In more recent times, philosophers like Wittgenstein and Davidson have enriched the cognitivist position in a variety of ways. It is suggested that no other species matches human beings in the complexity, rationality and sophistication of thought and no other species has language like the one we have; this uniqueness is possible only if we believe that it is only language that makes thought possible. The physical and mental activity of most animals is restricted to seeking food and sex, rearing their young ones and protecting themselves from predators. Even the most painstakingly trained dogs and cats display a finite number of responses and behaviour of any significant complexity. But human beings, in addition to all the above, do Physics **a**nd Mathematics, make ships and space shuttles of increasing complexity, explore genetic mappings, write poems and create music et. Some may argue that this is possible because of some abstract intelligence. But this according to the cognitive view is calling water H2O. It does not explain anything. It is language that makes thought possible. In any case, there is no doubt that language is extremely important for our thoughts. As Russell says, it is unnecessary to prolong the catalogue of the uses of language in thought. As compared to images, we produce words easily to articulate our thoughts and listen to them effortlessly to understand others. If we did not have words and sentences, abstract images will almost be impossible to comprehend. Language provides a stable system. Every time people say 'tree', they mean the same object, although their pronunciations may be significantly different. We always need words to recall or describe an image, a thought or an event in our memory.

It is the process of break-up of a language into varieties and sub-varieties, languages and sub-languages which leads to the situation of a number of different languages having many systematic resemblances. Such languages are described as a 'family' and just as members of a human family show varying degrees of relatedness, have specified relationships and have common ancestors at different generation levels and, ultimately, a common ancestor, so do languages. In this sense Indo-European is the 'ancestor 'of a very large number of languages spoken today in Asia and Europe. An interesting question is - why do languages continuously break up and fragment into different dialects, sub-dialects, varieties and other languages? The reason lies perhaps in the fact that so many persons, millions, in fact, speak a language under varying conditions and in different situations. This is the cause of what may be called natural change. Languages also start changing when they come in contact with each other. The third reason of language change is geographical division or separation. As a result of all this, the most self-evident fact about language is that people talk differently. This synchronic variation leads to historical change and change leads to split, a process which produces a number of languages related to each other in different degrees of closeness or aff**in**ity and constituting a family. However, we must bear in mind that a language is not born, nor does it die except when every single one of its speakers dies , as has happened to Etruscan, Gothic, Cornish, and a good many other languages. Hence the tetras family, ancestor, parent, and other genealogical expressions when applied to languages must be regarded as no more than metaphors. Languages are developments of older languages rather than descendants in the sense in which people are descendants of their forefathers".

One of the chief characteristics of human beings is their ability to communicate with their fellow beings. They convey complex messages concerning every aspect of life. A child even at a very early age learns to communicate by imitating the recurrent sound pattern s/he has become familiar with, It is only later that s/he learns the visual representation of speech i.e, writing. Even though most Indians can use at least two languages as a means of communication, they generally take speech for granted, and seldom stop to think about the complex speech mechanism that makes spoken language possible. The analysis of the spoken form of a language is by no means simple, because each of us uses an infinite number of speech sounds which combine in different ways to form the strings of words we utter. For the convenience of description and analysis therefore, we need to break up the strings of utterances into the smallest units of sounds possible. Phonetics is the study and science of speech sounds. When we produce speech sounds we use the Speech Mechanism which comprises of certain organs of the body, such as the muscles of the chest, the tongue, the lips and so on. The movement of these organs causes some disturbance which travels to the ear of the listener in the form of sound waves. The listener then interprets them as sounds. The organs of speech perform different functions in the production of speech. In other words, the air that we breathe is modified in different ways to result in various combinations of consonants and vowels. That is probably why Speech is popularly known as 'modified breathing. The organs of speech and their speech functions can be described with reference to three systems: the Respiratory System, the Phonatory System and the Articulatory System.

The organs of speech of which the Respiratory system is' comprised are the lungs, the muscles of the chest and the windpipe or trachea. The primary function of the lungs as we all know is to enable us to breathe or respire. The muscles of the chest expand and consequently the lungs expand and draw the outside air in. Thus we breathe in or inhale the air which passes through the trachea into the lungs. When the muscles of the chest contract the lungs are compressed and throw the air out. Thus we breathe out or exhale the air we have breathed in. The function of the Respiratory system is to provide the air stream which is the basis for the production of speech sounds. It is the air stream that acts as a source of energy and is modified by the speech organs as it passes in and out of the lungs during the normal course of breathing. Besides the lungs there are other organs that can also provide an air stream for the production of speech sounds e.g. the glottis and the velum. The glottalic-air stream is the air stream above the glottis which is closed for the production of sounds using this air stream. Similarly, sounds produced with the air in front of the velum or the soft palate. The passage of air from the lungs is blocked by the back of the tongue which makes firm contact with the velum. This is known as the velaric air stream. A large number of African languages and some South American languages make use of these air streams for the production of speech sounds. Of the Indian languages only Sindhi uses the glottalic air stream mechanism for the production of some speech sounds. But the pulmonic or lung air is used by all the languages of the world, and it is the air exhaled from the lungs which initiates the air stream. This air-stream mechanism is known as the pulmonic aggressive air stream mechanism when the air which is breathed initiates the air-steam for speech production. It is known as the pulmonic egressive air steam mechanism.

The Phonatory system is comprised of the larynx in the throat. As the air that comes out of the lungs it is modified before it meets the outside air. At first, the air is modified in the upper part of the trachea where the larynx is situated. The larynx is a muscular structure. The front part of this structure can be felt in the neck, and is commonly known as the 'Adam's apple'. The Adam's apple is more prominent in men than in women. As the vocal cords are separated at the back it is possible for them to assume a large number of positions. From among these we shall describe three important positions. The vocal cords can be held wide apart with a wide opening between them (the glottis). The air can pass freely through this opening without setting the vocal cords into vibration. This is the position of the vocal cords when we breathe. A large number of speech sounds are produced with the vocal cords in this position. During the production of these sounds we cannot hear any 'hum' when we plug our ears with our forefingers, or place the palm of the hand on the throat. They are called voiceless sounds. For example, the first sounds in the English words, sit, sheet, fever and think. The second position that the vocal cords can assume is one in which they are held loosely together. When they are in this position the pressure of air from the lungs makes them vibrate. The sounds produced when the vocal cords vibrate are called voiced sounds. During the production of these sounds we can hear the 'hum' we talked about earlier. For example, the consonant sounds underlined in the English words veil, these, zoo, me, nose. Whereas all English vowels are voiced, some English consonants are voiceless and some are voiced. The vocal cords can be held tightly together along their whole length. So that the glottis is closed and no air can escape through it. This is the position that the vocal cords take when we eat or drink to prevent food or liquid from entering the windpipe.

Vowel and Consonant are popularly defined with reference to the letters of die alphabet. Thus the letters a, e, i, o and u are called 'vowels' and the rest are 'consonants. This definition is misleading because 'vowel' and 'consonant' are essentially categories of speech sounds. When we label the letters of the alphabet as 'vowel' and 'consonant' we probably do tt on the basis of the assumption that there is perfect correspondence between die letters and the sounds they stand for. This correspondence, unfortunately, is not perfect in any language, least of all in English. 'Vowel' and 'Consonant' are also defined in phonetic and linguistic terms. When we define them in phonetic terms, we do so with reference to their production, when we define them in linguistic terms we refer to their function in a given language. In phonetic terms, a vowel is a sound for whose production the oral passage is unobstructed, so that the air can flow from the lungs to the lips and beyond without being stopped' without having to squeeze through a narrow construction, which would cause audible friction.

A consonant, on the other hand, is a sound for whose production the air current is completely stopped, or is forced through a narrow constriction which causes audible friction.

Let us look at a few examples. For instance die English word bar. The vowel represented by the letters ar, is produced with the mouth wide open and the tongue low in the mouth. The air passage is unobstructed and the air passes into the atmosphere outside without any friction. The sound is therefore called a vowel. The sound represented by the letter b is produced by a complete closure of the oral passage of air at the two lips, and then the sudden release of the air held behind the closure. Thus, there is an obstruction, for a while, to the flow of air from die lungs. Because o f this obstruction the resultant sound is regarded as a consonant.
