# Supplementary material for: Dataset for classifying English words into difficulty levels by undergraduate and postgraduate students
Source: Data Brief. 2023 Oct 31;51:109744. doi: 10.1016/j.dib.2023.109744 (PMC10661753; doi:10.1016/j.dib.2023.109744)
Supplement: Supplementary file 10 [file mmc10.docx]

What is Literary Criticism: This too is one of those awkward questions about which I have already told you. When someone asks you whether you like a particular book, a novel, a short story, and you say "yes" or "no" you are on the threshold of literary criticism. When s/he asks you why, and then you attempt an answer trying to rationalize your perception, you are "doing" literary criticism, albeit at a rudimentary level. Similarly, you do film or art criticism. Understanding and interpreting literary experience, even when not articulated can be literary criticism. Now, this can be an amateurish response. Alternatively, it could also be a highly sophisticated, professional one of the kind we generally read in review columns or journals.

English poets and critics whose works you read have battled over the question of the relative superiority or otherwise of criticism over creation: Wordsworth and Arnold, for example. Some enduring criticism has also come into being by way of "Defense of Poetry". You already know of some of these, such as Sidney's (1554-1586) and Shelley's (1792-1822) Essays. The classical criticism of the Greeks and Romans grew around attacks on and defense of the position of poets in a civil society. "Aesthetics" and "Poetics" were terms that were earlier used before the vogue of • "Criticism" set in. Very generally speaking,

A THEORY (any theory) provides a system by which experience can be organized and made sense of, or at least into something which will be comprehensible. All theories are constructed against the threat of chaos, which is the absence of system or organizing principles, to make sense of what comes to us, however, provisional and imperfect that sense may have to be. That is the basis of all dogma, religious and political alike.

Contradistinction

By definition then, you can see the difference between what is generally referred to as theory, and what we have always known as literary criticism. Theory, in the context of literature, is the set of broad assumptions about literature, and the function of criticism. When you say, for example, that economic condition of a society, its modes of production, and class system, in any given time determine the literature of that society in that given period of its history, you are making a theoretical pronouncement. However, when you start analyzing any work of art, a poem or a novel of that society and period from that theoretical perspective, you are doing literary criticism. This kind of "reading" of literature is, of course, known as Marxist criticism, that is literary criticism where you have applied the Marxist 'Theory" of literature. And, you may have done it yourself, without knowing it! "Reading" in this sense is purposive reading, with theory in mind. Literary criticism, you may say, thus, is applied theory.

Secondly, or to put the same thing in a different way, whereas the object of literary criticism is any given, particular, literary (oral or written) work, that of classical poetics or contemporary theory is usually generalization about literature. Invariably, it is the second which leads to the practice of the first. This indeed is what scientific method is all about: from the particular or individual case studies to the general; and For some reason, the term theory, as opposed to literary criticism, came into vogue when structuralism and post-structuralism opposed new criticism. In this sense, theory is used to describe a wide range of approaches from the Marxist to the deconstructionist school.

Indians from now on would be treated with suspicion irrespective of whether they were from the middle classes, the urban poor, villagers or the landed gentry. The term 'mutiny' with its wide ideological ramifications covered all aspects of British behaviour. The question is whether the adoption of English by Indians in the mean time had given a wrong mutinous orientation to the Indian middle classes in this phase responded to issues such as individual freedom differently from the way they ' were supposed to do by their masters. The reformist acts undertaken by the regime earlier may also have unleashed trends in India that drew inspiration from the spirit to: critique orthodoxy. With pioneers such as Ram Mohan Roy active in the realm of reform and change earlier in the century, various communities in India, forged ahead of others and threw up visionaries who would work with all their might to transform the environment at a latter day. Some of these communities took keen interest in education and aimed at ushering in the era of modernity. This second phase was especially productive in the area of literature unlike the previous one that had inspired only thinkers and reformers. The reason is not far to seek. With increase in the knowledge of English, a large number of creative minds began to look at their counterparts in England as their role models and sought to emulate their example. I suggest through this point the emergence of a phenomenon in which the Indian art forms had an interface with the European ones. The latter helped the Indian mind to wrestle free from the highly stylised and moralistic renderings of myths in the 1ndian' writing. However, this phase unhappily caused the marginalisation of those Indian forms that struggled to represent new experiences, dichotomies and disharmonies in Indian life. It certainly took the middle class minds away from some of the best trends emerging in Indian language literatures

While Grant was thinking of anglicising the native, the British educational policy was running on completely different principles. Before the Anglicist viewpoint on education was realised in actual practice, the colonial system of education was guided primarily by pragmatic considerations. The encouragement of ancient and medieval Indian learning by the British was based on the policy of neutrality regarding religious-cultural matters. The fear of rousing the religious sensitivities of the Indians precluded any attempt at conversion or indoctrination. In other words, the policy on noninterference in education was conditioned by the imperatives of sociopolitical control over the newly conquered territories. It will then be worthwhile to ask - what were the reasons for this crucial policy shift? What made the colonial masters think that the old policy of placating the native's cultural prejudices was outdated? Can we assume that the framers and supporters of the new educational policy redefined British imperial domination? A study of the various minutes and tracts of those who wanted to introduce English in India reveals that they differed from the old policy makers on the issue of controlling the socio-cultural life of their Indian subjects. They did not see imperialism as a purely political practice with minimum interference in the socio-cultural affairs of the colony. Imperialism was now constructed as a transcendent force sweeping the entire globe with a great burden of 'civilising' the natives. In other words Anglicists represent imperialism as a benevolent political enterprise zealously working towards the improvement of less developed communities. The assumption, of course, is that these communities cannot improve on their own due to the innate depravity/ inferiority of their socio-cultural practices. Let us examine the new developments that were to take place in Indian society as a result of this grand assumption.

**RAJA RAMMOHUN ROY’S LETTER TO AMHERST**

The Letter is the most significant indigenous contribution to the debate of education in the early nineteenth century. Roy wrote this Letter in order to protest against the ruling regime's decision to open a Sanskrit College in Calcutta. It has been already pointed out that before the new educational policy was implemented, the government of India supported instruction in the classical Indian languages. It funded institutions involved in the spread of Hindu and Mohammedan learning and gave financial assistance to Sanskrit and Arabic scholars in order to encourage them to achieve, distinction in their respective fields. The government also provided grants for the printing of ancient Sanskrit and Arabic texts in the original and in translation. The Governor Generals in the formative years of British rule were committed to the spread and encouragement of native learning. Both Warren Hastings and Lord Minto were instrumental in the establishment of many institutions where oriental literature was taught. By 1823 this official policy of the government faced repeated assaults from a number of quarters. The attack primarily came from the missionaries, the Anglicists of the East India Company and the 'enlightened' Bengali elite. The proposal for the establishment of a Sanskrit College at Calcutta was a part of this decision. Roy's Letter locates a contradiction between the modem, scientific spirit of European civilisation and its educational policy in India. The proposal to establish the new Sanskrit College comes as a shock to Roy since, in his view, it does not conform to the liberal image of the British. It is clear from a careful perusal of the letter that Roy expects the colonial power to be true to its liberal image and find ways and means to promote western sciences, and arts for the benefit of its Indian subjects.

**THE REGIONALISATION SCHEME AND ITS CRIITIQUE**

The Commission also recommended "the development of all modern Indian languages for use in education as well as in administration...." But the most important recommendation of the Commission was the regionalisation of the medium of education in the Universities. This proposal drew a lot of protest from scholars, educationists and academics. The protest was primarily directed at what was seen as an attempt to create eradicable barriers in the way of national unity. Attempts were made to remind the Indian State that the political union of the country had a relatively short history and India in the past never achieved a perfect unity due to the multiplicity of languages and cultures. It was feared by scholars like A A Kane that if the Commission's plan of regionalisation was implemented:

All hope of India's making rapid progress as a modern country and sinking its regional and linguistic differences in the interest of national unity would be shattered. (Source)

If national unity is to be kept at the top of any educational agenda what will be the solution to the language problem in India? Kane like many educationists of the day does not pay any attention to the idea of a federal language evolved by the Radhakrishoan Commission. English, in Karve's opinion is best suited to occupy the hallowed place of the medium of instruction as it has been not only used "as the language of instruction during the last one hundred years", but "has also served us as a means of communication between scholars from different parts of India and between them and the intellectuals of the outside world." (Source)

British ideologues instilled into the native mind that English was an agent of rationality and modern sensibility. The average middle class Indian student well versed in English in post-independence, India was inclined to enroll him in the 'original' centres of English learning (i.e. Oxford and Cambridge) than to languish in `sub-standard' institutes of higher education in India. The association of English with civilisational and cultural progress was never seriously questioned even after Independence. The bureaucracy and universities of Independent India have shown a marked preference for students who have received education in Western countries thus replicating the pre-independence period when the England-returned elite manned the higher echelons of administration and educational institutions. The Commission failed to see all these factors responsible for the so-called brain drain. Though it did not take suitable measures to counter the bias for English education, it certainly strengthened the dominant notion of the superiority of English by recommending the establishment of major universities.

It is also certain that these universities would not have posed a significant challenge to Oxford or Harvard, given the attitude of the Indian elite towards `foreign' education. Furthermore the domination of these foreign-returned Indians over Indian bureaucracy and academic institutions would not be questioned as long as the notorious notion of the superiority of western educational practices exists. As Kamat points out: *Only a determined policy of encouraging Indian scholarship coupled with the creation of more and better facilities for advanced research in the country and a careful selection and strict control of research students sent abroad for training could eventually break this unhealthy grip of the foreign returned elite on the academic life of the country. (Source)*

RISING SOCIAL DISPARITIES AND THE NEW EDUCATIONAL POLICY

The Kothari Commission was conscious of the link between higher education and rising unemployment. It discovered that the universities produced a great number of graduates, many of who were unable to find employment in the job market. Yet it could not frame a policy to help the educated unemployed youth. Its measures to counter unemployment were negative in nature. It sought the implementation of the selective admission system so that overproduction of graduates could be prevented. Thus the 1970s saw an increase in urban and rural unemployment. The social disparities gave rise to an intense dissatisfaction of the Indian masses with the political establishment. As the vast majority of the population toiled for its daily bread education became the first casualty. The decline of education in the backward and rural areas of India reflected disillusionment with the educational system, which did not equip the people with any source of livelihood. The National Policy on Education (NPE) of 1986 reflected a growing realization on the part of the ruling establishment that educational goals had to be redefined. The NPE did not worry about the decline of academic standards. Rather it stressed: ***The need for the removal of disparities and emphasised the steps to be taken to equalize educational opportunities by attending to the specific needs of those who have been denied equality so far — women, scheduled castes and tribes. (Source)*** However, the NPE's quest for social justice remained incomplete as it maintained a status quo on the question of English in India. On the subject of languages it observed that:

***The Educational policy of 1968 had examined the question of the development of languages in great detail; its essential provision can hardly be improved upon and are as relevant as today. (Source)***

Let us look at the political scene in India in the nineteen fifties, the decade that followed Independence. Two things particularly lurked at that time in the minds of most Indians. The first was the happening of Partition, both on the western and the eastern sides of the country. No well-meaning serious individual could overlook the occurrence and go on one's safe and smooth way, gifted as it would have been by one's economic background. The second was Independence itself, a result of close to hundred years of effort and struggle by countless Indians. The free India required what could be called nation building, an act-positive in nature and designed to meet the basic requirements of all and inspired by the humanist urge to plan things `idealistically'. Unlike other species, human beings have the capability to rise above themselves and think of goals bigger than the individual ones. The values and principles standing at the back of the Indian National Movement testified clearly to this capability of human beings, more so in the case of those who fought relentlessly against the mightiest power in the world in the nineteenth and the twentieth centuries. To see this is to know that a newly emerged nation had its future secure in the progressive and humanistic modern perspective.

There was, however, a problem with this vision, pulled in the two different directions of pain and despair (for the reason of Partition) on one side and the potentiality of humanist endeavour supportive of Nationalist values on the other. The problem related to the presence of the privileged in our midst. The lobbies, active all along the National Movement were those of the industrialists and the urban rich. These expressed themselves in what was called moderate politics, the politics of compromise, bargain and circumspection.

As far as the literary writing in English by an Indian writer goes, one notices a great deal of versatility there. This is accomplished by a skill and acumen difficult to achieve ordinarily. Large international publishing houses sponsor these writers and project them as representative voices of great merit. The contemporary Indian writer in English makes a great splash one day, drawing attention to him/ her for the artistic feat achieved. The appeal of this writer is assessed commercially with mention of the number of copies sold of a particular work. The 'signing amount' of the work in question is also big news with publicity of the work arranged proportionate to the money changing hands on the big occasion. Thus, literary writing in English, particularly with reference to India, has become more of a commodified entity meant to be packaged for the market and sold across the counter all over the globe to rake in huge profits. Artistic value of such a commodity, which also happens to be a literary work, is a mere by-product. Interestingly, this by-product, too, is also made to add value to the primary commodity through the organised effort of the publicity industry that newspapers, magazines, journals, prize-giving organisations and academic centres of high learning in the western world have finally become. English in India is part of a tricky world, a globalised atmosphere run on the hard logic of commercialism.

For a 3^rd^ world country, the phenomenon of the rise and growth of English in India is indeed educative. It offers a great many insights into the likely shifts and changes one notice in historical epochs. It also makes clear to us the role played by power centres in the creations of paradigms, canons and perspectives, many of which we have been witness to in our eventful history of the last hundred and fifty years. Today, we have a burgeoning middle class fed on the ideas of merit, efficacy and skill that English is capable of imparting in the modern world.
